# Systematic functional identification of cancer multi-drug resistance genes

**DOI:** 10.1186/s13059-020-1940-8

**Published:** 2020-02-07

**Authors:** Man-Tat Lau, Shila Ghazanfar, Ashleigh Parkin, Angela Chou, Jourdin R. Rouaen, Jamie B. Littleboy, Danielle Nessem, Thang M. Khuong, Damien Nevoltris, Peter Schofield, David Langley, Daniel Christ, Jean Yang, Marina Pajic, G. Gregory Neely

**Affiliations:** 1 The Dr. John and Anne Chong Lab for Functional Genomics, Charles Perkins Centre and School of Life & Environmental Sciences, The University of Sydney, Sydney, NSW 2006 Australia; 2 Genome Editing Initiative, The University of Sydney, Sydney, NSW 2006 Australia; 3 School of Mathematics and Statistics, The University of Sydney, Sydney, NSW 2006 Australia; 4 The Judith and David Coffey Life Lab, Charles Perkins Centre, The University of Sydney, Sydney, NSW 2006 Australia; 5 The Kinghorn Cancer Centre, The Garvan Institute of Medical Research, 384 Victoria St, Darlinghurst, Sydney, NSW 2010 Australia; 6 The University of Sydney, Sydney, NSW 2006 Australia; 7 Garvan Institute of Medical Research, Darlinghurst, Sydney, NSW 2010 Australia; 8 St Vincent’s Clinical School, Faculty of Medicine, UNSW Sydney, Kensington, Sydney, NSW 2010 Australia

## Abstract

**Background:**

Drug resistance is a major obstacle in cancer therapy. To elucidate the genetic factors that regulate sensitivity to anti-cancer drugs, we performed CRISPR-Cas9 knockout screens for resistance to a spectrum of drugs.

**Results:**

In addition to known drug targets and resistance mechanisms, this study revealed novel insights into drug mechanisms of action, including cellular transporters, drug target effectors, and genes involved in target-relevant pathways. Importantly, we identified ten multi-drug resistance genes, including an uncharacterized gene *C1orf115*, which we named *Required for Drug-induced Death 1* (*RDD1*). Loss of *RDD1* resulted in resistance to five anti-cancer drugs. Finally, targeting RDD1 leads to chemotherapy resistance in mice and low *RDD1* expression is associated with poor prognosis in multiple cancers.

**Conclusions:**

Together, we provide a functional landscape of resistance mechanisms to a broad range of chemotherapeutic drugs and highlight RDD1 as a new factor controlling multi-drug resistance. This information can guide personalized therapies or instruct rational drug combinations to minimize acquisition of resistance.

## Background

Although many cancers can be treated with chemotherapeutic and targeted drugs, patients frequently develop resistance over time leading to disease relapse and poor prognosis. A basic functional understanding of genes and mechanisms involved in anti-cancer drug resistance can lead to new biomarkers, drug combinations, or patient-specific therapies. Pharmacogenomic profiling of cancer cell lines (CCL) [1–3] compares drug response to gene expression and has provided insights into anti-cancer drug mechanisms of action (MoA). Direct mechanistic interpretation of these data sets can be difficult [3], and functional genomics approaches can help elucidate drug MoA and resistance.

## Results and discussion

### Whole genome CRISPR knockout screens for 27 anti-cancer drugs

**Fig. 1:**
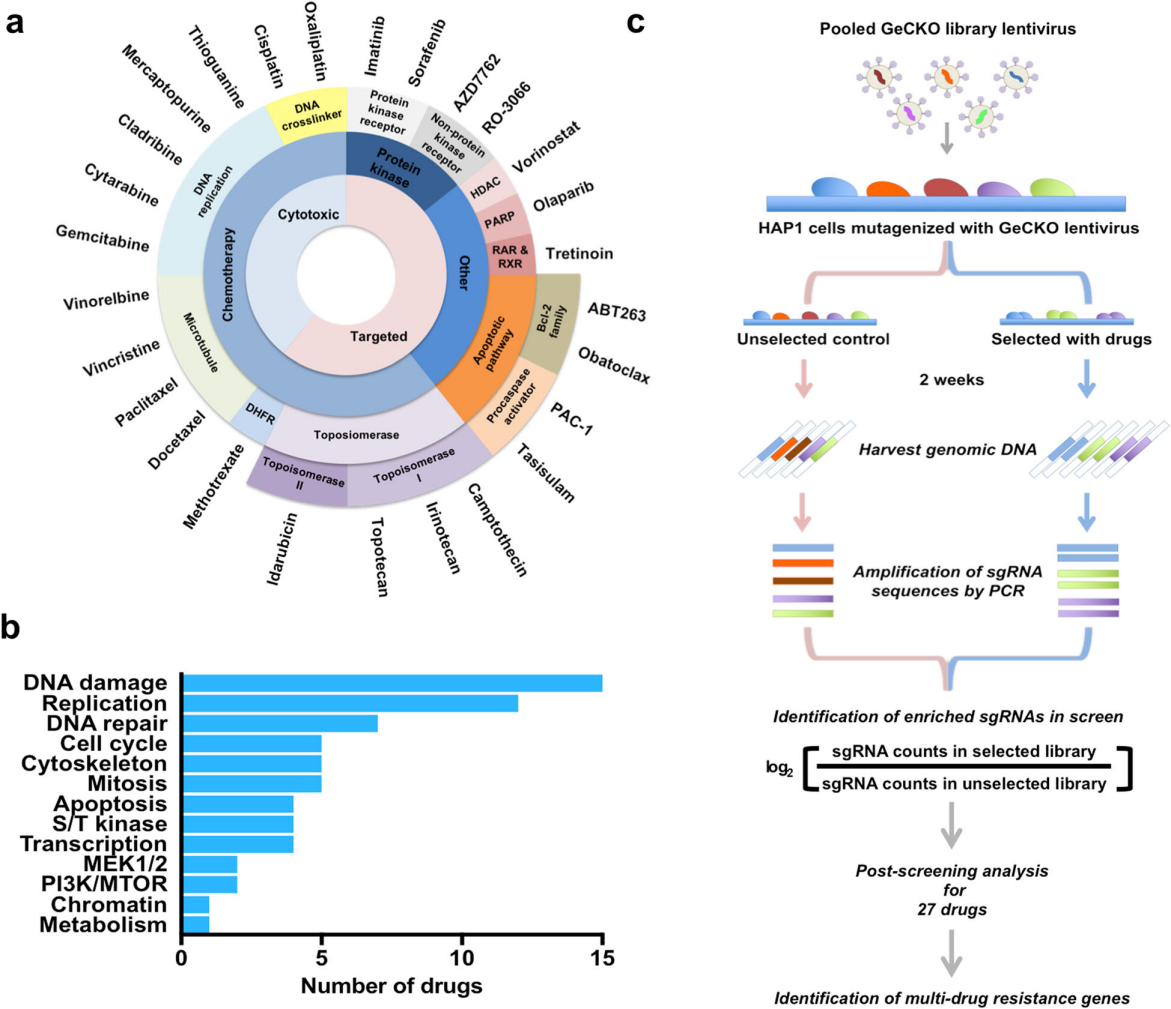
A CRISPR-Cas9 knockout screen identified genes required for the cytotoxicity anti-cancer drugs. **a** The panel of 27 screened drugs are classified based on their therapeutic targets and mechanisms of action. **b** Drugs are grouped according to their primary target/effector pathways and cellular functions. A single drug may be included in multiple categories. **c** Schematic design of pooled CRISPR library screens to identify the genes involved in drug sensitivity

Whole genome loss-of-function screens using the CRISPR-Cas9 system are an effective tool for identifying cell death or resistance mechanisms in response to anti-cancer drugs [4–8], bacterial toxins [9], or viral infection [10]. To generate a global perspective on resistance mechanisms that regulate sensitivity to anti-cancer drugs, we performed large-scale functional resistance screens to a spectrum of anti-cancer drugs, covering a wide range of targeted and cytotoxic agents in clinical use or preclinical development (Fig. 1a and Additional file 1: Table S1). The drugs used in this screen target various critical biological processes that are perturbed during cancer development and progression (Fig. 1b and Additional file 1: Table S1). We used the haploid cell line HAP1, a well-characterized model for functional genomic studies [11–15], and generated dose-response cell death curves for all drugs screened using a resazurin-based cell viability assay (Additional file 2: Figure S1). We mutagenized cells with the human Genome-scale CRISPR Knockout (GeCKO) v2 Library, a large-scale loss-of-function library consisting of 123,411 unique single guide RNA (sgRNA) sequences targeting 19,050 human genes [16]. Cells were selected for resistance using a minimal lethal concentration (IC90-99; Additional file 1: Table S1) of each anti-cancer agent for the first 3 days, and then lowered to allow recovery and expansion of resistant cells. Drug-resistant cells were recovered, and sgRNA abundance was quantified relative to an unselected control population (Fig. 1c, Additional file 3: Table S2). We then identified hits that were significantly enriched (false discovery rate [FDR] < 0.1) using the Model-based Analysis of Genome-wide CRISPR-Cas9 Knockout (MAGeCK) method [17]. From this, we found screens for 20 of 27 compounds yielded at least 1 significant hit (FDR < 0.1). For the 7 compounds (imatinib, olaparib, obatoclax, PAC-1, paclitaxel, RO-3066, and sorafenib) that did not yield significant data, this is likely due to screening at high drug concentrations (Additional file 4: Table S3). To evaluate our overall approach with respect to what has been previously reported for HAP1 cells, we compared the list of essential genes (i.e., dropout screen data; Additional files 3 and 5: Table S2 and S4) obtained using the GeCKO v2 library alongside HAP1 screening results obtained using the pLCV2-TKOv3 and pLCKO-TKOv3 gRNA libraries [18]. We observed substantial overlap between these three sets of essential genes (Additional file 2: Figure S2a). The set of 376 essential genes identified in our study include fundamental biological processes with enrichment of ribosome, aminoacyl-tRNA biosynthesis, RNA transport, RNA polymerase, pyrimidine metabolism, spliceosome, cell cycle, proteasome, DNA replication, and ribosome biogenesis in eukaryotes (Additional files 1, 2, and 5: Figure S2b, Table S4 and S5).

### Functional resistance profiles reveal known MoA

**Fig. 2:**
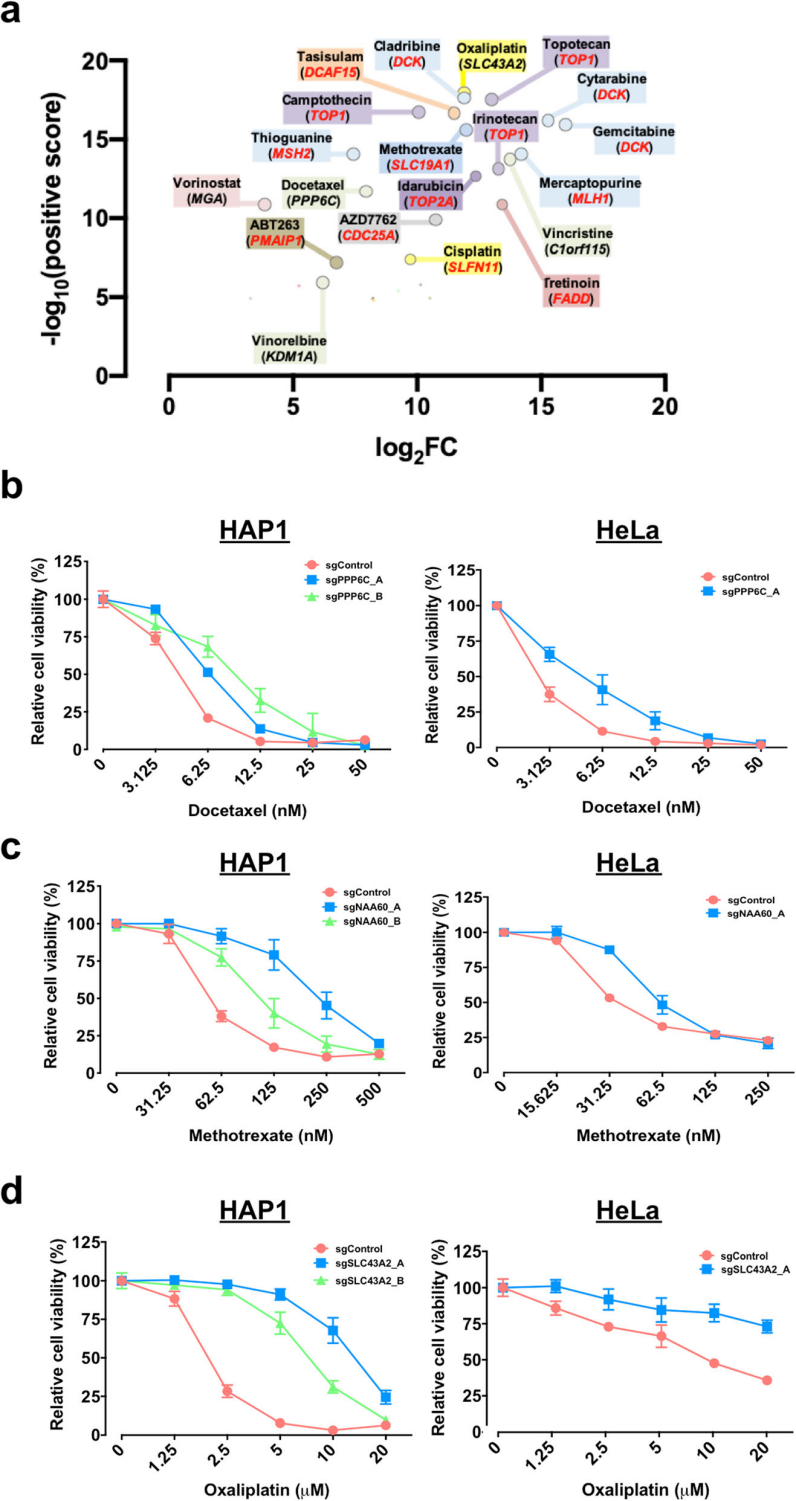
Whole genome resistance profiling identifies known and novel targets/mechanisms of action. **a** The top hit genes (FDR < 0.1) identified from each drug screening using MAGeCK. A subset of genes previously linked to modulate drug sensitivity (red text). **b** A CRISPR-Cas9 knockout screen identified PPP6C required for docetaxel cytotoxicity. CRISPR-Cas9-targeted cells decreased their sensitivity to docetaxel in HAP1 and HeLa cells. **c** NAA60 is involved in methotrexate sensitivity. CRISPR-Cas9-targeted cells decreased their sensitivity to methotrexate in HAP1 and HeLa cells. **d** A putative small-molecule transporter (SLC43A2) for oxaliplatin. CRISPR-Cas9-targeted cells decreased their sensitivity to oxaliplatin in HAP1 and HeLa cells

To assess the ability of this system to identify drug resistance genes or mechanisms, we compared our results with well-characterized drug MoAs. In resistance screens for various topoisomerase I inhibitors (camptothecin, irinotecan, and topotecan), the top ranked hit was *TOP1*, whereas conversely, the top ranked hit for the topoisomerase II inhibitor (idarubicin) was *TOP2A* (both FDR < 0.005, Fig. 2a, Additional files 2, 3, and 5: Figure S3a-f, Table S2 and S4). We also uncovered critical regulators involved in drug processing. For example, deoxycytidine kinase (encoded by *DCK*) which phosphorylates nucleoside analogues intracellularly to exert cytotoxicity [19] was the top ranked hit for the nucleoside analogues cladribine, cytarabine, and gemcitabine (FDR < 0.005, Fig. 2a, Additional files 2, 3, and 5: Figure S3 g-k, Table S2 and S4).

Whole genome resistance profiling effectively captured known cancer drug transport mechanisms. For instance, methotrexate (MTX, an anti-folate chemotherapeutic drug) mimics natural folates to block thymidine biosynthesis via inhibition of dihydrofolate reductase (DHFR) and requires a transporter to enter the cell. In our system, sgRNAs targeting the folate carrier *SLC19A1* showed strong enrichment for MTX resistance, independently validating this gene association from a prior study [20] (FDR = 0.00248, Fig. 2a, Additional files 2, 3, and 5: Figure S4a, Table S2 and S4).

Functional resistance profiling could also identify encoded proteins involved in drug target-relevant pathways. For example, resistance to AZD7762, a checkpoint kinases (CHKs) inhibitor, could be achieved through loss of the downstream CHK1 target, CDC25A (FDR = 0.00495, Fig. 2a, Additional files 2, 3, and 5: Figure S4b, Table S2 and S4). CHKs are important enzymes that regulate either the G1/S or the G2/M transition in the cell cycle. In response to DNA damage or incomplete DNA replication, CHKs become activated and transiently delay cell cycle progression to allow DNA repair or the completion of DNA replication*.* AZD7762 drives checkpoint abrogation via inhibition of CHK1, which stabilizes CDC25A and impairs DNA repair resulting in tumor cell death [21, 22] (Additional file 2: Figure S4b). Moreover, using pathway analysis, we were able to identify mismatch repair (MMR) machinery (such as *MLH1*, *MSH2*, and *MSH6*) (FDR < 0.005, Additional files 1, 2, 3, and 5: Figure S4c, Table S2, S4 and S6) from functional resistance screening for mercaptopurine (6-MP) and thioguanine (6-TG). This is consistent with known resistance mechanisms for these compounds [23, 24].

### Functional resistance profiles reveal novel MoA

In addition to known regulators of drug sensitivity, numerous top ranked resistance genes identified here have not been previously linked with drug MoA or resistance, such as *C1orf115*-vincristine, *KDM1A*-vinorelbine, *MGA*-vorinostat, *PPP6C*-docetaxel, and *SLC43A2*-oxaliplatin (all FDR < 0.005, Fig. 2a, Additional files 3 and 5: Table S2 and S4). *PPP6C*, previously implicated in tumorigenesis and progression [25, 26], was confirmed to regulate sensitivity to the microtubule inhibitor docetaxel (Fig. 2b). Within the top resistance genes for each compound, we also identified *N*-alpha-acetyltransferase 60 (NAA60, encoded by *NAA60*) (FDR = 0.00248; Additional files 3 and 5: Table S2 and S4), and this was validated as a mediator of methotrexate resistance (Fig. 2c). NAA60 is an N-terminal acetyltransferase that acetylates met-lys, met-ala, met-val, and met-met, and is required for normal chromosome segregation [27].

We also identified *SLC43A2*, a putative oxaliplatin transporter [28], as a major mediator of oxaliplatin cytotoxicity (FDR = 0.00495, Fig. 2a, Additional files 3 and 5: Table S2 and S4). *SLC43A2* encodes an L-type amino acid transporter (LAT4) which facilitates the movement of bulky neutral amino acids across the cell membrane [29]. We confirmed that knockout of *SLC43A2* reduced oxaliplatin sensitivity in multiple cell types (Fig. 2d), flagging *SLC43A2* as a potential key mediator of oxaliplatin cytotoxicity.

Tasisulam, an acyl-sulfonamide inhibitor, suppresses proliferation in a variety of human cancers [30]. Functional profiling for tasisulam resistance identified two significant hits, *DCAF15* and *DDA1*, which belong to the core subunits of the cullin-ubiquitin ligase complex [31] (both FDR = 0.00248, Additional files 2, 3, and 5: Figure S5a and b, Table S2 and S4). To confirm a role for *DCAF15* and *DDA1* in tasisulam cytotoxicity, we generated the relevant CRISPR-knockout cells. Depletion of *DCAF15* or *DDA1* resulted in an increased resistance to tasisulam in both HAP1 and HeLa cells (Additional file 2: Figure S5c and d). Our results independently confirm recent data implicating anti-cancer sulfonamides induce cell death by disrupting precursor mRNA splicing via the cullin-ubiquitin ligase-dependent degradation of RBM39/CAPERalpha [32, 33] (Additional file 2: Figure S5e).

**Fig. 3:**
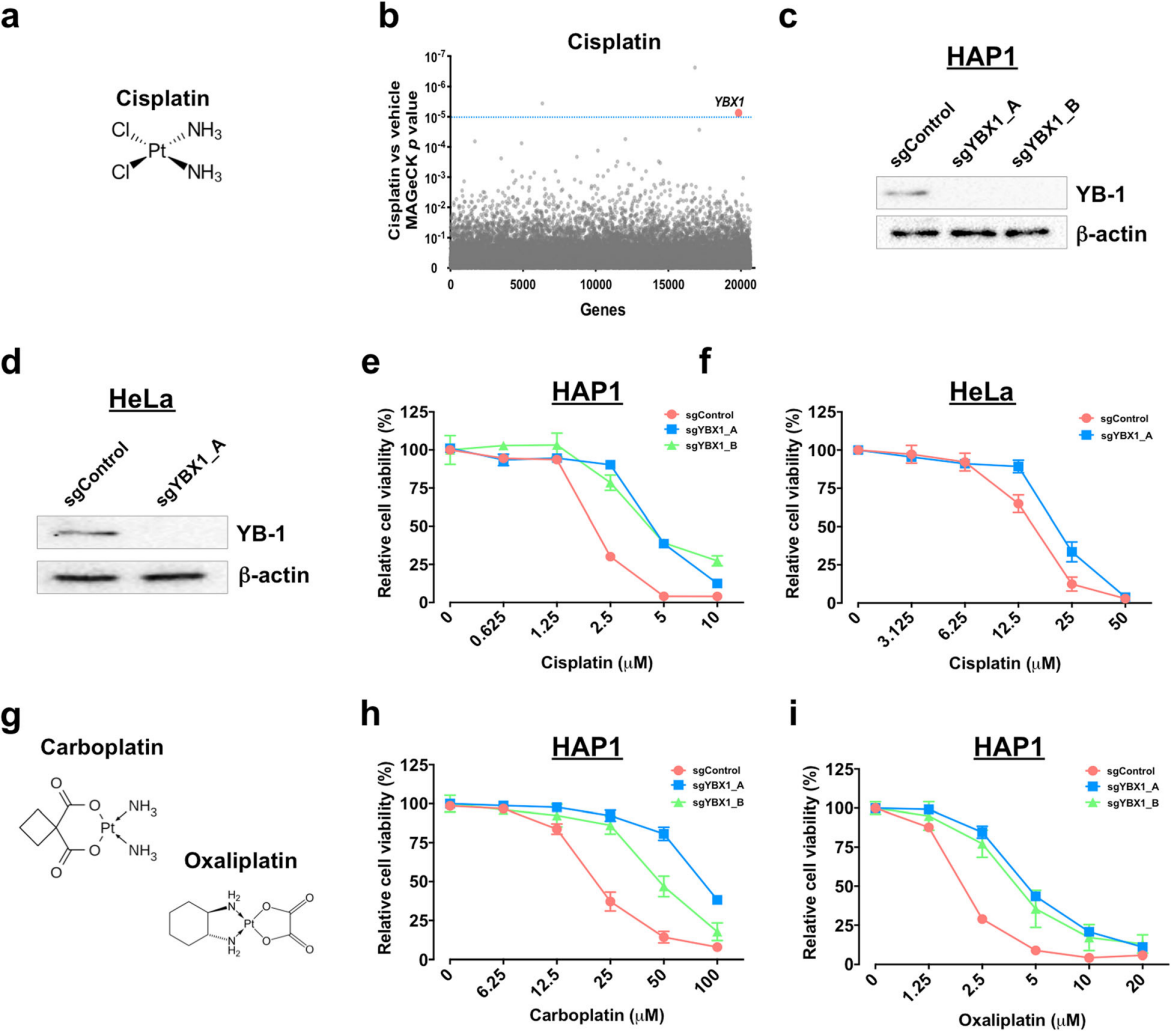
Depletion of YB-1 results in resistance to platinum-based drugs. **a** Chemical structure of cisplatin. **b** Enriched genes identified from screens for cisplatin. FDR = 0.1 (blue dotted line). **c** Western blot validation of sgRNA-mediated depletion of YB-1 in HAP1. **d** YB-1 and beta-actin levels were analyzed by Western blot analysis in CRISPR-Cas9-targeted HeLa cells. **e**, **f** CRISPR-Cas9-targeted cells decreased their sensitivity to cisplatin in **e** HAP1 and **f** HeLa cells. **g** Chemical structure of carboplatin and oxaliplatin. **h**, **i** Depletion of YB-1 in HAP1 cells reduced their sensitivity to **h** carboplatin and **i** oxaliplatin

Our whole genome resistance profiling also highlighted that the transcription factor Y-box-binding protein 1 (YB-1, the protein encoded by *YBX1*) was involved in sensitivity to cisplatin (FDR = 0.05116, Fig. 3a, b, Additional files 3 and 5: Table S2 and S4), and these results were independently validated (Fig. 3c–f). Cisplatin is a platinum-based genotoxic agent that blocks DNA replication by DNA crosslinking, induction of double-stranded DNA breaks, leading to cellular apoptosis [34]. Accordingly, YB-1-depleted cells displayed common resistance to other platinum-based drugs, carboplatin and oxaliplatin (Fig. 3g–i), in agreement with recent evidence that checkpoint adaptation (entry into mitosis with damaged DNA) is likely a primary pathway in cisplatin-induced cell death at pharmacological concentrations [35]. Together, our findings reveal new genetic insight into drug-specific and broad mechanisms behind therapeutic resistance, while creating a comprehensive data set that can guide further investigation into new resistance mechanisms.

### Identification of novel multi-drug resistance gene *C1orf115*/*RDD1*

**Fig. 4:**
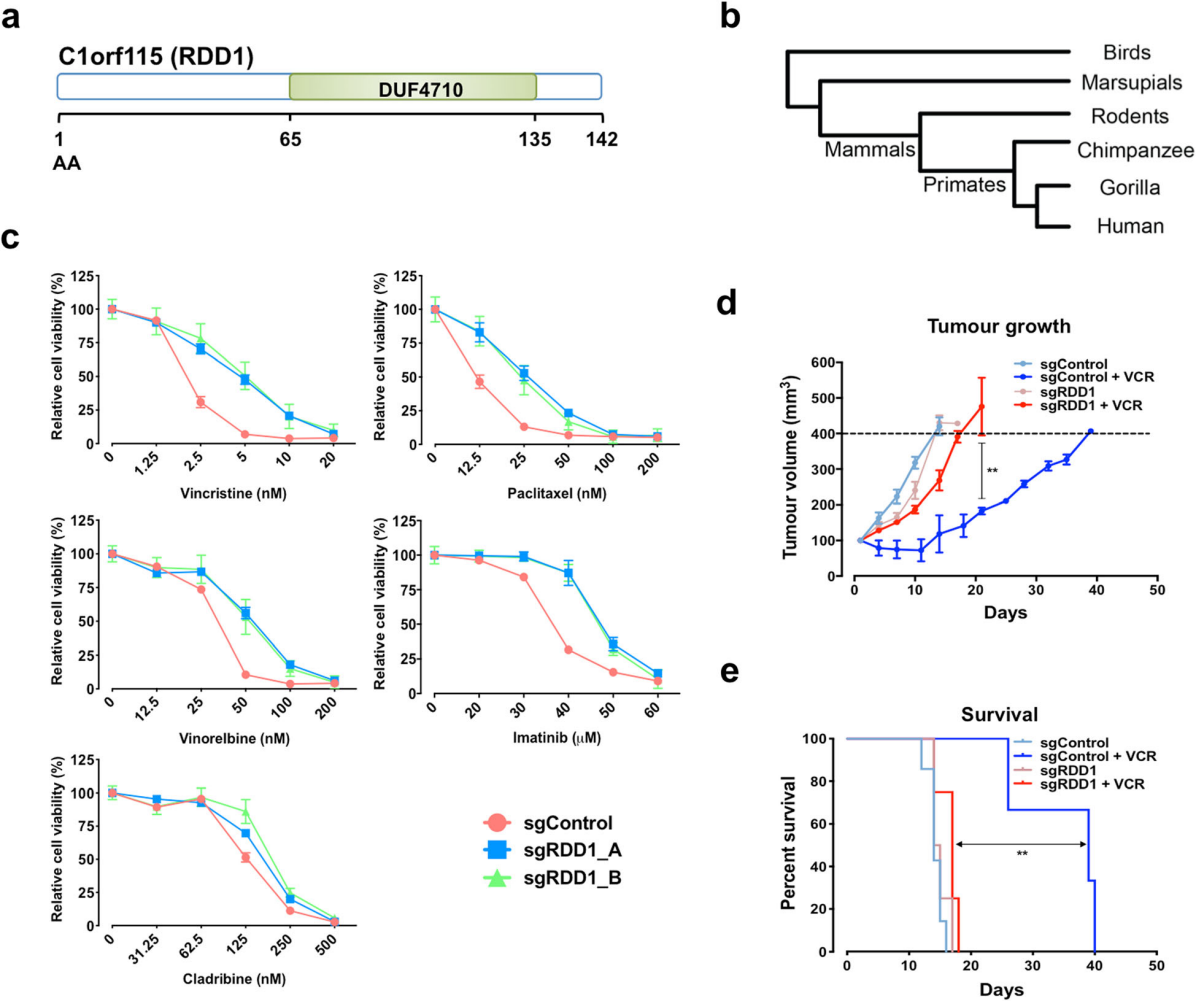
Characterization of *RDD1.***a** Schematic protein structure of RDD1. **b** Gene tree of *RDD1* from birds to human. **c** CRISPR-Cas9 *RDD1*-targeting cells decreased their sensitivity to the drugs (as indicated) in HAP1 cells. **d**, **e** CRISPR-Cas9 *RDD1*-targeting HeLa cells decreased their sensitivity to vincristine (VCR) in xenograft tumor model. **d** Tumor growth and **e** overall survival are shown. All data represented as mean ± SEM (*n* = 4–6 mice per group). **d** One-way ANOVA followed by Tukey’s post hoc test or **e** log-rank test, **p < 0.01

In total, our screening isolated 56 significant chemotherapy resistance genes across the 20 compounds tested (FDR < 0.1, Additional file 5: Table S4). We next investigated genes that when targeted confer resistance to more than 1 drug (multi-drug resistance; MDR). In total, we identified 10 genes that were significantly enriched with resistance to 2 or more drugs (FDR < 0.1, Additional file 1: Table S7). Also, MDR genes were classified by their primary cellular roles. A subset of these MDR genes has previously been linked to general cellular cytotoxicity or previously implicated in cancer drug resistance. Among these are pro-apoptotic genes (including *BAX* and *PMAIP1* [also known as *NOXA*]), *DCAF15*, deoxycytidine kinase (*DCK*), mismatch repair genes (including *MLH1*, *MSH2*, and *MSH6*), topoisomerase I (*TOP1*), and topoisomerase IIA (*TOP2A*). Importantly, we identified a novel uncharacterized MDR gene, *C1orf115* (named here *Required for Drug-induced Death 1* (*RDD1*), Fig. 4a), which we further investigated.

*RDD1* encodes a previously uncharacterized protein containing a DUF4710 domain of unknown function (Fig. 4a). This gene is widely conserved in vertebrates, including mammals, marsupials, and birds (Fig. 4b). In humans, RDD1 is broadly expressed with highest expression in the gastrointestinal tract, brain, and female reproductive system [36]. We identified RDD1 as an MDR gene that, when targeted, causes significant resistance to two anti-cancer drug screens: cladribine (FDR = 0.00099) and vincristine (FDR = 0.00495), and guides targeting this gene were also highly enriched (albeit not significant) in imatinib, paclitaxel, and vinorelbine resistance screens (Additional files 2, 4, and 5: Figure S6, Table S3 and S4). We confirmed our screening results and found that targeting RDD1 with two distinct sgRNAs (Additional file 2: Figure S7a) resulted in resistance to these five anti-cancer drugs, primarily anti-tubulin agents (paclitaxel, vincristine, and vinorelbine; Fig. 4c, Additional file 2: Figure S7b and c). Similar results were obtained independently using RNA interference, confirming targeting RDD1 leads to multi-drug resistance (Additional file 2: Figure S7d and e). Moreover, loss of RDD1 conferred resistance to vincristine and vinorelbine (which prevent microtubule polymerization [37]) and paclitaxel (which stabilizes microtubules by preventing depolymerization [38]), and this resistance could be rescued by overexpression of a gRNA-resistant RDD1 construct (Additional file 2: Figure S7f). Microtubule-targeting drugs are clinically used to control various malignancies, especially ovarian and breast adenocarcinomas [39]. Further, loss of RDD1 also conferred resistance to vincristine or paclitaxel in vivo (Fig. 4d, e and Additional file 2: Figure S7 g) resulting in increased tumor growth and shortened lifespan in drug-treated animals. Of note, targeted RDD1 cells did not cause broad resistance as sensitivity to AZD7762, BI2536, cabazitaxel, docetaxel, mercaptopurine, obatoclax, and vorinostat was retained (Additional file 2: Figure S8). Collectively, our data highlights RDD1 as a new central regulator of anti-cancer drug sensitivity and loss of RDD1 as an important mediator of multi-drug resistance.

### Low *RDD1* expression was associated with poor prognosis in multiple cancers

**Fig. 5:**
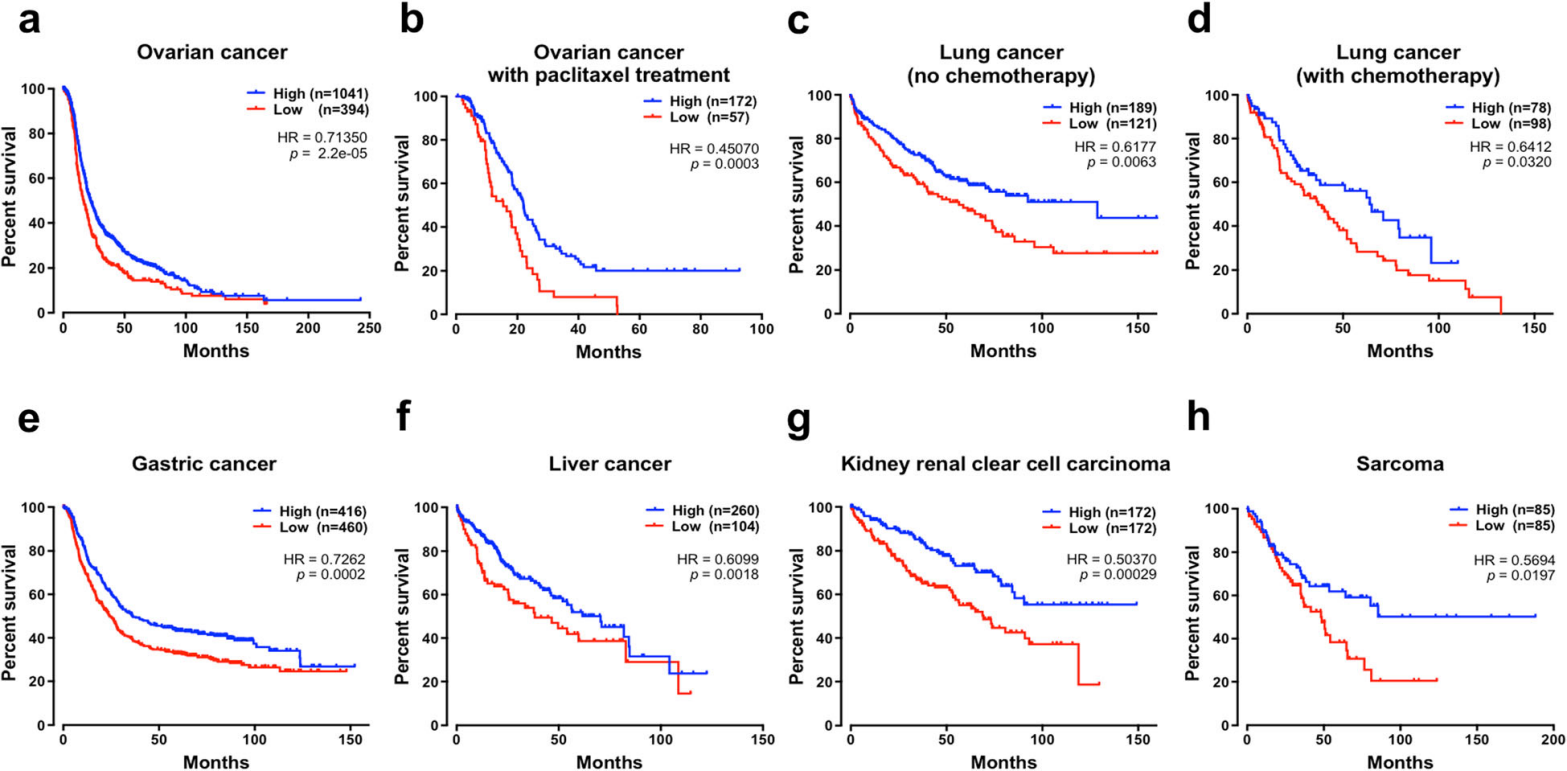
Low *RDD1* expression was associated with poor prognosis in multiple cancers. **a**–**h** The Kaplan-Meier survival plots of patient overall survival using the **a**–**f** Kaplan-Meier Plotter database or **g**, **h** OncoLnc online tools. *p* values were calculated using the log-rank (Mantel-Cox) test

To assess the prognostic relevance of *RDD1*, we next evaluated RDD1 expression compared to cancer patient outcome [40]. Notably, significant reductions in *RDD*1 mRNA expression were observed in multiple cancers such as breast cancer, colorectal cancer, lung cancer, and ovarian cancer (normal versus tumor) (Additional file 1 and Additional file 2: Figure S9a, b and Table S8). Moreover, low *RDD1* expression was significantly associated with poor patient survival (*n* = 1435 with *p* = 2.2e−05 and hazard ratio of 0.7135) in ovarian cancer patients (Fig. 5a), specifically in a paclitaxel-treated cohort (Fig. 5b). This effect was further replicated in a second independent patient cohort (Additional file 2: Figure S10a, b). *RDD1* expression was significantly correlated with patient outcome in lung cancer (*p* = 0.0063 and hazard ratio of 0.6177, Fig. 5c; *p* = 0.032 and hazard ratio of 0.6412, Fig. 5d), gastric cancer (*p* = 0.0002 and hazard ratio of 0.7262, Fig. 5e), liver cancer (*p* = 0.0018 and hazard ratio of 0.6099, Fig. 5f), kidney renal clear cell carcinoma (*p* = 0.00029 and hazard ratio of 0.5037, Fig. 5e), and sarcoma (*p* = 0.0197 and hazard ratio of 0.5694, Fig. 5f). Together, these data highlight the relevance of *RDD1* expression in controlling cancer drug sensitivity and link low RDD1 expression with reduced patient survival in various cancers.

Our results demonstrate the value of systematic functional identification of anti-cancer resistance genes and MoAs of small compounds. From a biological perspective, our genome-wide CRISPR screen established new functional gene/drug interactions that may help us understand how anti-cancer drugs kill tumors and how resistance inevitably develops. This includes functional validation of *RDD1*, and other new anti-cancer drug resistance genes warrant further characterization. This study thereby provides the scientific community with a comprehensive multi-dimensional dataset and functional framework for future experimental and computational investigations. Together these data, complemented by existing drug sensitivity information [2, 3] and CRISPR-Cas9 knockout data [41], may help to shape personalized therapies, instruct future drug development, and guide the design of molecularly optimized combination treatments for cancer patients.

## Materials and methods

### Cell culture

Human HAP1 cells were generously provided by Dr. Thijn R. Brummelkamp [11]. HeLa cells were gifted from Dr. Adam R. Cole (Garvan Institute). HEK293T cells were obtained from the ATCC. Cells were cultured in medium (HAP1 cells in IMDM (Sigma-Aldrich); HeLa and HEK293T cells in DMEM (Sigma-Aldrich)), containing 10% bovine calf serum (BCS; Hyclone Laboratories), 1× GlutaMAX, 100 U/ml penicillin G, and 100 g/ml streptomycin (Thermo Fisher Scientific) in a humidified atmosphere of 5% CO_2_–95% air at 37 °C. HeLa and HEK293T cell lines were authenticated using STR DNA profiling. HAP1 cells were not formally authenticated. All cell lines tested negative for mycoplasma.

### Cell viability assay

Trypsinized cells (1.5 × 10^4^ cells; unless stated otherwise) were seeded in each well of a 96-well plate. After 24 h, various concentrations of anti-cancer drugs were added, and the cells were incubated for an additional 72 h. After incubation, the medium was aspirated from each well and 150 μl of fresh medium containing a 0.002% solution of resazurin (Sigma-Aldrich) was added to the wells and incubated for 4 h at 37 °C. The absorbance was measured at 570 nm using a microplate spectrophotometer (FLUOstar Omega, BMG Labtech).

### Lentivirus production

To generate lentivirus, the human lentiCRISPRv2 plasmid library (Addgene 1000000048) was co-transfected with packaging plasmids pCAG-VSVG and psPAX2 (Addgene plasmids 35616 and 12260, respectively). Briefly, a T-75 flask of 80% confluent HEK293T cells was transfected in OptiMEM (Thermo Fisher Scientific) using 8 μg of the lentiCRISPRv2 plasmid library, 4 μg pCAG-VSVG, 8 μg psPAX2, 2.5 μg pAdVantage (Promega), 30 μl of P3000 Reagent (Thermo Fisher Scientific), and 30 μl of Lipofectamine 3000 (Thermo Fisher Scientific). Cells were incubated overnight, and then, the media were changed to DMEM (Sigma-Aldrich) with 10% BCS and 1× GlutaMAX (Thermo Fisher Scientific). After 48 h, viral supernatants were collected and centrifuged at 2000 rpm for 10 min to get rid of cell debris. The supernatant was filtered through a 0.45-μm ultra-low protein binding filter (Merck Millipore). Aliquots were stored at − 80 °C.

### Cell transduction using the GeCKO v2 library

HAP1 cells were transduced with the GeCKO v2 library by spinfection. Briefly, 2 × 10^6^ cells per well were plated into a 12-well plate in IMDM media supplemented with 10% BCS and 8 μg/ml polybrene (Sigma-Aldrich). A titrated virus was added in each well along with a no-transduction control. The plate was centrifuged at 2000 rpm for 1 h at 37 °C. After the spin, cells were incubated overnight and then enzymatically detached using TrypLE™ Express (Thermo Fisher Scientific). Cells were counted, and each well split into duplicate wells. One replicate was treated with 1 μg/ml puromycin (Thermo Fisher Scientific) for 3 days. Percent transduction was determined as cell count from the replicate with puromycin divided by cell count from the replicate without puromycin multiplied by 100. The virus volume yielding a MOI (multiplicity of infection) of approximately 0.4 was used for large-scale screening.

### HAP1 anti-cancer drug resistance screen

1 × 10^8^ HAP1 cells were transduced as described above using 12-well plates with 2 × 10^6^ cells per well. Puromycin was added to the cells 24 h post-transduction and maintained for 7 days. Cells were pooled together into larger flasks after 3 days incubation of puromycin. On day 7, cells were split into treatment conditions in duplicate with a minimum of 2.5 × 10^7^ cells per replicate (seeding density ~ 225,000 cells/cm^2^). Two replicates were cultured in IMDM supplemented with anti-cancer drugs (Additional file 1: Table S1), and one replicate was cultured in regular IMDM media. Replicates were either passaged, or fresh media with drugs was added every 2–3 days. During screenings, we reduced the concentration of the drugs to select the resistance cells in CRISPR KO pool whereas untransduced HAP1 cells were treated with the drugs to ensure the drug was cytotoxic in each case. Cells were harvested after 14 days of the treatment for genomic DNA analysis.

### Genomic DNA sequencing

Genomic DNA (gDNA) was extracted from harvested cells with a Blood & Cell Culture Midi Kit (Qiagen) and used for PCR reactions as described previously [16]. Primers used to amplify lentiCRISPR v2 sgRNAs for the first PCR are as follows: sense, 5′-AAT GGA CTA TCA TAT GCT TAC CGT AAC TTG AAA GTA TTT CG-3′, and antisense, 5′-TCT ACT ATT CTT TCC CCT GCA CTG TTG TGG GCG ATG TGC GCT CTG-3′.

A second PCR was performed to attach Illumina adaptors and barcode samples. The second PCR was done in a 100-μl reaction volume using 5 μl of the first PCR product. Primers for the second PCR include both a variable length sequence to increase library complexity and a 6-bp barcode for multiplexing of different biological samples: sense, 5′-AAT GAT ACG GCG ACC ACC GAG ATC TAC ACT CTT TCC CTA CAC GAC GCT CTT CCG ATC T (1–9 bp variable length sequence) (6 bp barcode) tct tgt gga aag gac gaa aca ccg-3′, and antisense, 5′-CAA GCA GAA GAC GGC ATA CGA GAT AAG TAG AGG TGA CTG GAG TTC AGA CGT GTG CTC TTC CGA TCT tct act att ctt tcc cct gca ctg t-3′.

Amplification was carried out with 18 cycles for the first PCR and 24 cycles for the second PCR. PCR products from the second PCR were gel extracted, quantified, mixed, and sequenced using a HiSeq 2500 (Illumina). The sgRNA sequences against specific genes were recovered after removal of the tag sequences using the Checkout script (http://100bp.wordpress.com) and Cutadapt (ver. 1.12).

Enrichment of sgRNAs (Additional file 3: Table S2), genes (Additional files 4 and 5: Table S3 and S4), and KEGG pathways (Additional file 1: Table S6) was analyzed using MAGeCK [17] (ver. 0.5.7) by comparing read counts from cells after drug selection with counts from matching unselected cell population.

### Plasmid constructs and gene validation

To validate the candidate genes from screening, sgRNAs from the parent library were cloned into pLentiCRISPRv2 (Addgene plasmid 52961). The control sgRNA was used from the parent library. Lentiviruses were produced as described above, and transduced HAP1 or HeLa cells were selected with 1 μg/ml puromycin 24 h post-infection. Two weeks later, puromycin was removed, and cells were allowed to recover for three additional days before analysis. Gene disruption efficiency was verified by Western blot. The sequences of the sgRNAs used are in Additional file 1: Table S9.

The gRNA-resistant construct (OE_RDD1) was made by directed mutagenesis using the Quick-Change kit (Stratagene) following the manufacturer’s protocol. To create the OE_RDD1 construct, the sgRNA targeting site (sgRDD1_B; Additional file 1: Table S9) on pLenti-C-mGFP (Origene) containing human C1orf115 gene (pLenti-C1orf115-mGFP) was changed to gRNA resistance sequence (synonymous mutations) using the following oligonucleotide—sense: 5′-GCC GCT TAT AGC GCT CCT TTC GCT GTA GCC ACC AGC GTG GTA TCC-3′, and anti-sense: 5′-AGC GAA AGG AGC GCT ATA AGC GGC AGC GAA GCC TTG CAG GCC G-3′.

### Western blot analysis

YB-1 antibody was purchased from Cell Signaling Technology, Inc. Beta-actin antibody was purchased from Abcam.

Cells were harvested in lysis buffer (50 mM Tris (pH 7.5), 150 mM NaCl, 1% NP40, 0.5% sodium deoxycholate, 1 mM EDTA, and 0.1% SDS) containing protease inhibitor cocktail (Sigma-Aldrich), and the protein concentrations were determined using the BCA Protein Assay (Thermo Fisher Scientific). The proteins (20 μg) were electrophoresed on 10% SDS-polyacrylamide gels, transferred to PVDF membranes (Amersham Bioscience), and incubated with specific primary antibodies at 4 °C overnight. After washing, the membranes were incubated with horseradish peroxidase-conjugated secondary antibodies for 1 h and were then visualized with enhanced chemiluminescent substrate (Thermo Fisher Scientific).

### Reverse transcription quantitative real-time PCR

Total RNA was prepared using TRIzol reagent (Invitrogen) according to the manufacturer’s instructions. Single-stranded cDNA was synthesized from 2 μg total RNA according to the manufacturer’s procedure (Bio-Rad). The primers used for SYBR Green RT-qPCR were as follows: for human RDD1, sense 5′-AGT ACG GCA AGA ATG TCG GG-3′ and anti-sense 5′-TTA GCG CAC GAA GGA TAC CA-3′; for GAPDH, sense 5′-ATG GAA ATC CCA TCA CCA TCT T-3′ and anti-sense 5′-CGC CCC ACT TGA TTT TGG-3′. RT-qPCR was performed using the Roche LightCycler 480 II Real-Time PCR System equipped with a 384-well optical reaction plate. Relative quantification of mRNA levels was performed using the comparative Cq method (∆∆Cq method) with GAPDH as the reference gene.

### siRNA transfections

siRNA transfections were performed using Lipofectamine RNAiMAX Reagent (Invitrogen) following the manufacturer’s protocol. Briefly, 5 × 10^5^ cells were seeded into 6-well tissue culture plates 1 day prior to transfection with 50 nM RDD1 siRNA (siRDD1_1-hs.Ri.C1orf115.13.1 and siRDD1_2-hs.Ri.C1orf115.13.2) or with a non-targeting control siRNA (siControl) (Integrated DNA Technologies). After 48 h, the cells were seeded in each well of a 96-well plate for cell viability assay as described above.

### Xenograft experiments

Survival studies were performed by subcutaneously injecting 1 million HAP1 or HeLa cells (sgControl or sgRDD1) resuspended in 50% matrigel and 50% PBS, into immunocompromised Balb/c-Fox1nuAusb (mude) mice. Treatment commenced when tumors reached 150mm^3^ (100%), and mice were randomized into a treatment group: (a) saline control (intraperitoneally, twice weekly (day 1, day 4)), (b) vincristine (VCR; 0.5 mg/kg intraperitoneally, once weekly), and (c) paclitaxel (PTX; 20 mg/kg, intraperitoneal injection, twice weekly (day 1, day 4)). Tumor size was monitored twice weekly by calipers, and tumor volume calculated using the formula: 0.5 × length × width^2^. Animals were euthanized at ethical endpoint (tumor volume > 400%), and tissues collected for analyses. Survival analyses were performed using the log-rank test on *n* = 4–6 mice per group.

### Patient survival analysis

We performed survival analysis for RDD1 using the Kaplan-Meier Plotter (http://kmplot.com/analysis) and OncoLnc (www.oncolnc.org) online tools that base their analysis on publicly available gene-expression datasets such as GEO (Affymetrix microarrays only) and TCGA [42].

### Statistical analysis

Statistical analysis performed was specified in the figure legends. *p* < 0.05 was considered statistically significant, unless stated otherwise. No corrections for multiple testing were made for the MAGeCK hits. No statistical methods were used to determine the sample size before starting experiments. Cell biology experiments were not randomized, and the investigators were not blinded with regard to sample allocation and evaluation of the experimental outcome. For xenograft experiments, blinding and randomization were performed. Statistical analysis was performed using GraphPad Prism (V7.0.1, GraphPad) and R ver. 3.5.2.

## Supplementary information

**Additional file 1: Table S1.** This table contains the drug information used for screens. **Table S5.** This table contains enriched KEGG pathways for essential genes. **Table S6.** This table contains enriched KEGG pathways from screens. **Table S7.** This table contains a list of gene hits and scores for MDR genes. **Table S8.** This table contains the oncomine data for Additional file: Fig. S9b. **Table S9.** This file contains sgRNA sequences used for gene validation.

**Additional file 2: Figure S1.** Drug response curve for all the 27 screened drugs in HAP1 cells. **Figure S2.** CRISPR dropout screening identifies HAP1 essential genes. **Figure S3.** Functional resistance profiles identify known MoA. **Figure S4.** Functional resistance profiles reveal known MoA. **Figure S5.** Loss of DCAF15 or DDA1 leads to tasisulam resistance. **Figure S6.** Identification of novel multi-drug resistance gene *C1orf115*/ *RDD1*. **Figure S7.** Loss of RDD1 results in multiple drug resistance. **Figure S8.** CRISPR-Cas9 targeted RDD1 cells do not display broad resistance in HeLa cells. **Figure S9.** RDD1 expression in human cancers. **Figure S10.** Low *RDD1* expression is significantly associated with poor patient survival.

**Additional file 3: Table S2.** This table contains sgRNA level data after MAGeCK analysis.

**Additional file 4: Table S3.** This table contains drug screens data that does not pass the FDR < 0.1 cutoff.

**Additional file 5: Table S4.** This table contains a list of gene hits and scores after MAGeCK analysis.

**Additional file 6.** Review history.

## Data Availability

Screening raw data are available at SRA by referencing the BioProject number PRJNA601000 [43]. Results of drug CRISPR screens are provided in the Additional files. All other datasets generated during this study are available from the corresponding authors upon reasonable request.
