# Supplementary material for: Systematic functional identification of cancer multi-drug resistance genes
Source: Genome Biol. 2020 Feb 7;21:27. doi: 10.1186/s13059-020-1940-8 (PMC7006212; doi:10.1186/s13059-020-1940-8)
Supplement: Supplementary file 2 — Additional file 2: Figure S1. Drug response curve for all the 27 screened drugs in HAP1 cells. Figure S2. CRISPR dropout screening identifies HAP1 essential genes. Figure S3. Functional resistance profiles identify known MoA. Figure S4. Functional resistance profiles reveal known MoA. Figure S5. Loss of DCAF15 or DDA1 leads to tasisulam resistance. Figure S6. Identification of novel multi-drug resistance gene C1orf115/ RDD1. Figure S7. Loss of RDD1 results in multiple drug resistance. Figure S8. CRISPR-Cas9 targeted RDD1 cells do not display broad resistance in HeLa cells. Figure S9. RDD1 expression in human cancers. Figure S10. Low RDD1 expression is significantly associated with poor patient survival. [file 13059_2020_1940_MOESM2_ESM.pdf]

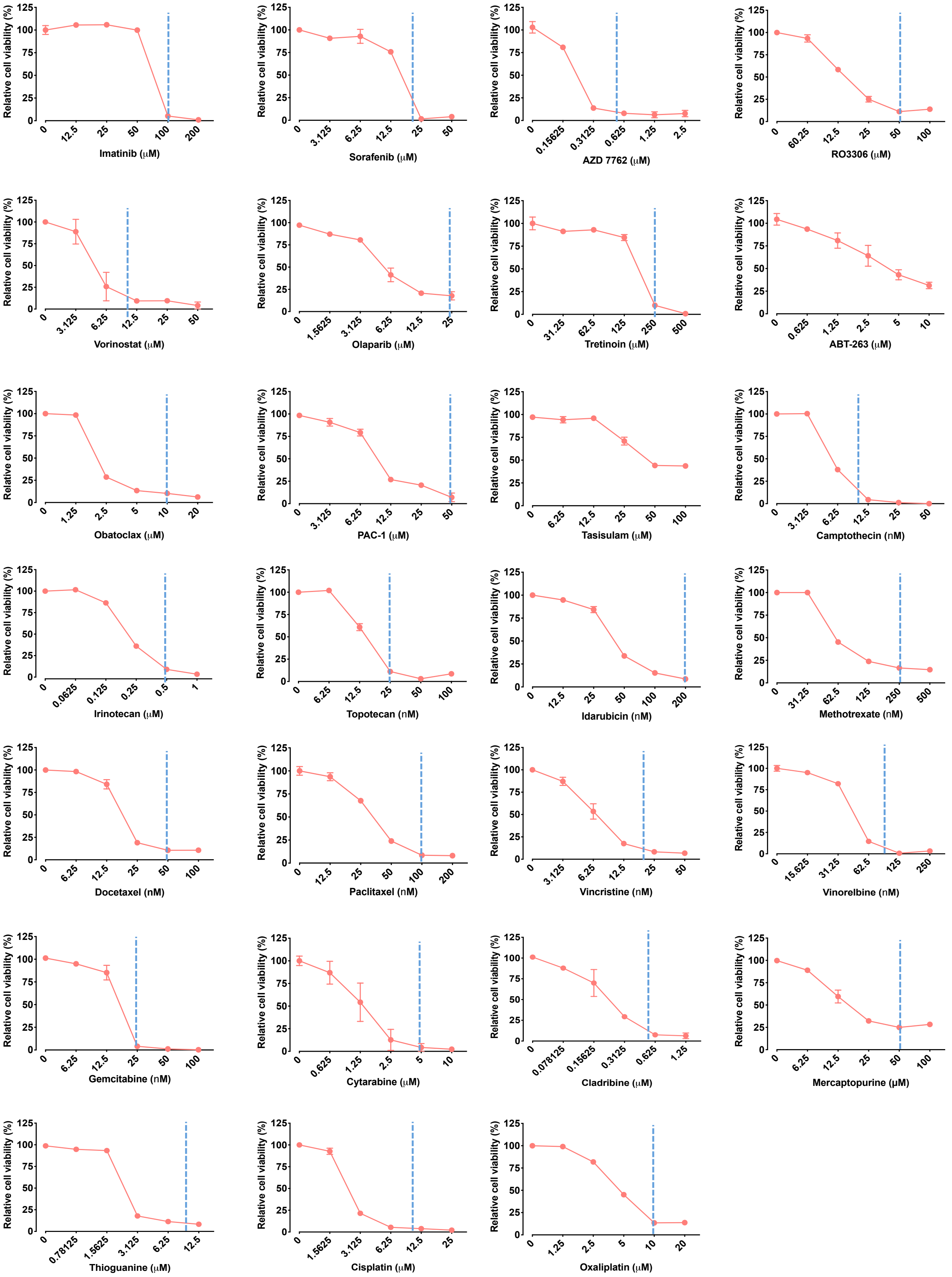
Relative cell viability (%)
0 25 50 75 100 125
0 12.5 25 50 100 200
Imatinib (μM)
0 3.125 6.25 12.5 25 50
Sorafenib (μM)
0 0.15625 0.3125 0.625 1.25 2.5
AZD 7762 (μM)
0 60.25 12.5 25 50 100
RO3306 (μM)
0 3.125 6.25 12.5 25 50
Vorinostat (μM)
0 1.5625 3.125 6.25 12.5 25
Olaparib (μM)
0 31.25 62.5 125 250 500
Tretinoin (μM)
0 0.625 1.25 2.5 5 10
ABT-263 (μM)
0 1.25 2.5 5 10 20
Obatoclax (μM)
0 3.125 6.25 12.5 25 50
PAC-1 (μM)
0 6.25 12.5 25 50 100
Tasisulam (μM)
0 3.125 6.25 12.5 25 50
Camptothecin (nM)
0 0.0625 0.125 0.25 0.5 1
Irinotecan (μM)
0 6.25 12.5 25 50 100
Topotecan (nM)
0 12.5 25 50 100 200
Idarubicin (nM)
0 31.25 62.5 125 250 500
Methotrexate (nM)
0 6.25 12.5 25 50 100
Docetaxel (nM)
0 12.5 25 50 100 200
Paclitaxel (nM)
0 3.125 6.25 12.5 25 50
Vincristine (nM)
0 15.625 31.25 62.5 125 250
Vinorelbine (nM)
0 6.25 12.5 25 50 100
Gemcitabine (nM)
0 0.625 1.25 2.5 5 10
Cytarabine (μM)
0 0.078125 0.15625 0.3125 0.625 1.25
Cladribine (μM)
0 6.25 12.5 25 50 100
Mercaptopurine (μM)
0 0.78125 1.5625 3.125 6.25 12.5
Thioguanine (μM)
0 1.5625 3.125 6.25 12.5 25
Cisplatin (μM)
0 1.25 2.5 5 10 20
Oxaliplatin (μM)

Fig. S1

a

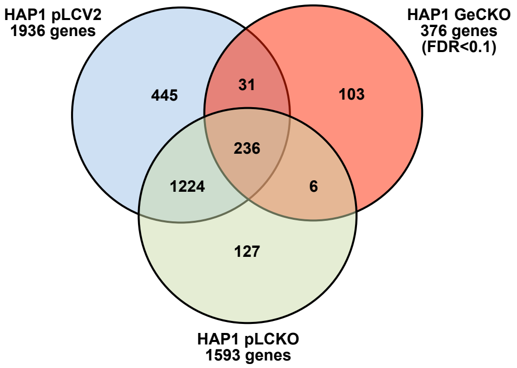
HAP1 pLCV2
1936 genes
HAP1 GeCKO
376 genes
(FDR<0.1)
445
31
103
236
1224
6
127
HAP1 pLCKO
1593 genes

b

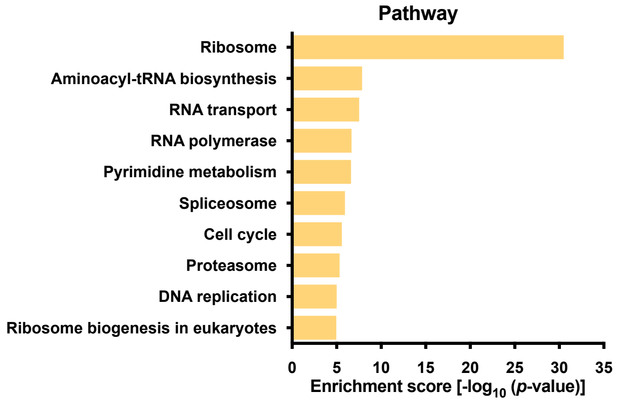
Pathway
Ribosome
Aminoacyl-tRNA biosynthesis
RNA transport
RNA polymerase
Pyrimidine metabolism
Spliceosome
Cell cycle
Proteasome
DNA replication
Ribosome biogenesis in eukaryotes
0
5
10
15
20
25
30
35
Enrichment score [-log10 (p-value)]

Fig. S2

a

Drug name	Drug Target	Gene Hit	Rank
Camptothecin	Topoisomerase I	*TOP1*	1
Irinotecan	Topoisomerase I	*TOP1*	1
Topotecan	Topoisomerase I	*TOP1*	1
Idarubicin	Topoisomerase II	*TOP2A*	1

g

Drug name	Drug Target	Gene Hit	Rank
Cladribine	DNA synthesis	*DCK*	1
Cytarabine	DNA synthesis	*DCK*	1
Gemcitabine	DNA synthesis	*DCK*	1

b

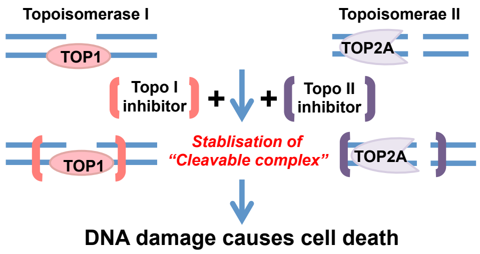

h

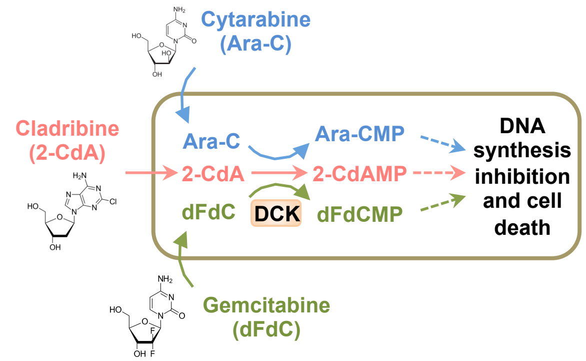

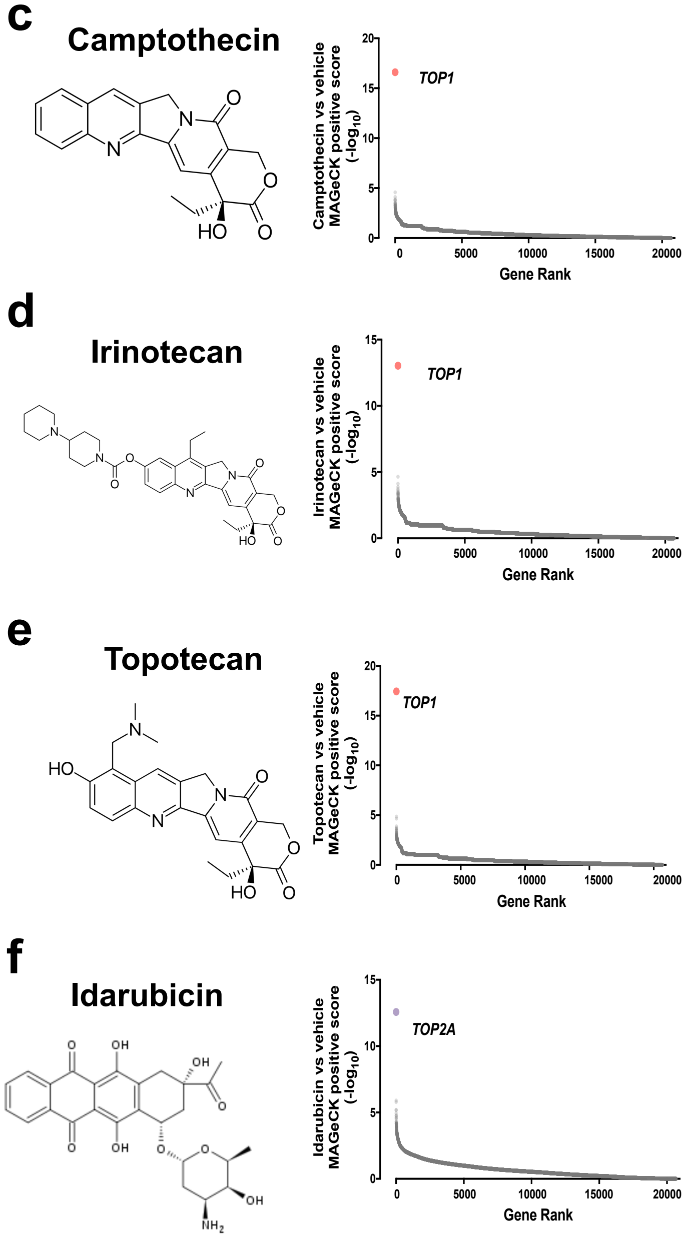

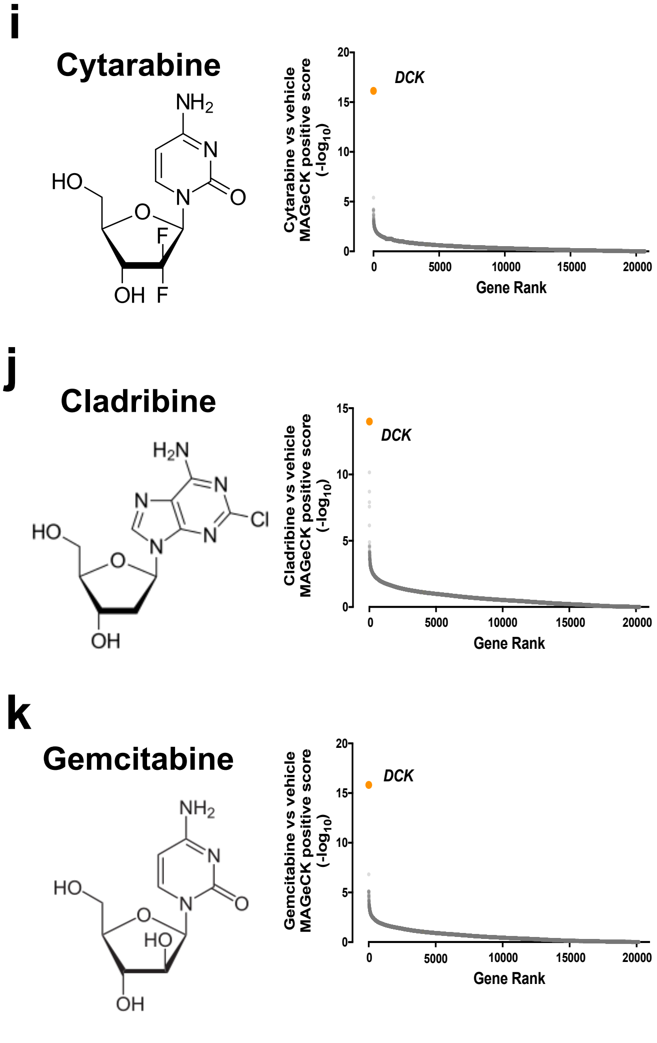

Fig. S3

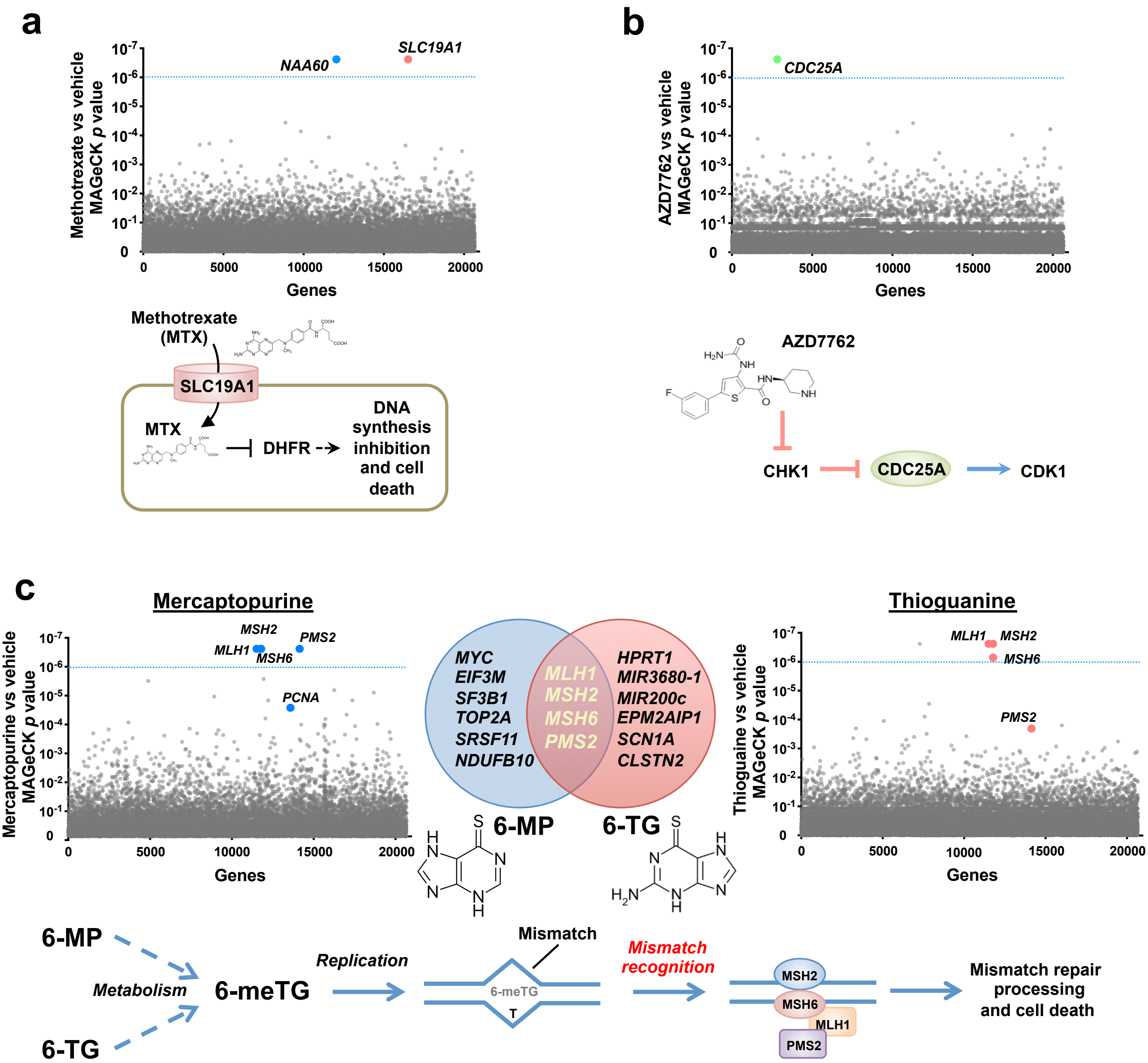
a
Methotrexate vs vehicle MAGeCK p value
NAA60
SLC19A1
Genes
Methotrexate (MTX)
SLC19A1
MTX
DHFR
DNA synthesis inhibition and cell death
b
AZD7762 vs vehicle MAGeCK p value
CDC25A
Genes
AZD7762
CHK1
CDC25A
CDK1
c
Mercaptopurine
Mercaptopurine vs vehicle MAGeCK p value
MSH2
PMS2
MLH1
MSH6
PCNA
Genes
MYC
EIF3M
SF3B1
TOP2A
SRSF11
NDUFB10
MLH1
MSH2
MSH6
PMS2
HPRT1
MIR3680-1
MIR200c
EPM2AIP1
SCN1A
CLSTN2
6-MP
6-TG
Thioguanine
Thioguaine vs vehicle MAGeCK p value
MLH1
MSH2
MSH6
PMS2
Genes
6-MP
Metabolism
6-TG
6-meTG
Replication
Mismatch
6-meTG
T
Mismatch recognition
MSH2
MSH6
MLH1
PMS2
Mismatch repair processing and cell death

Fig. S4

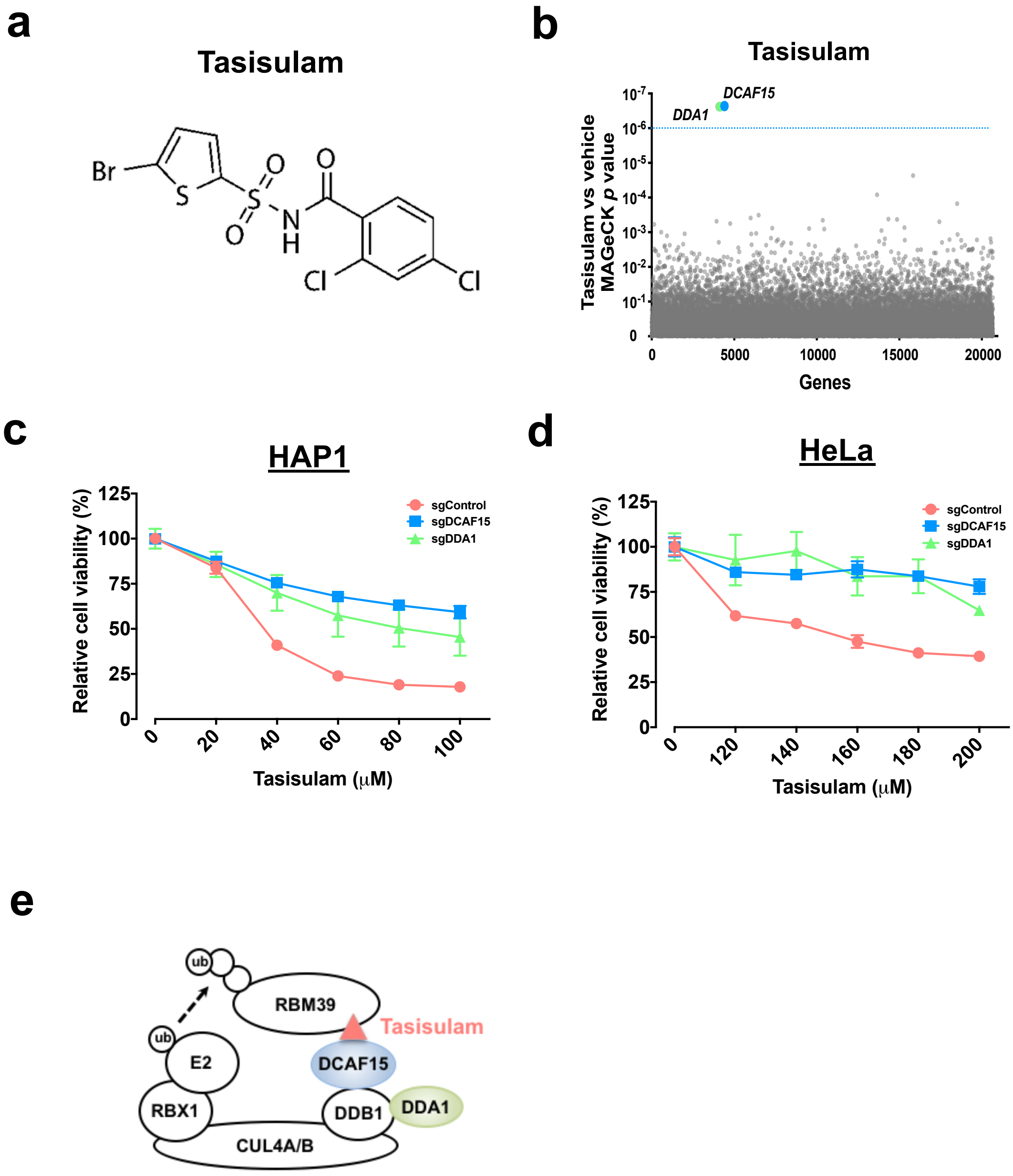
a
Tasisulam
Br
S
O
O
O
N
H
Cl
Cl
b
Tasisulam
Tasisulam vs vehicle MAGeCK p value
DCAF15
DDA1
Genes
c
HAP1
Relative cell viability (%)
sgControl
sgDCAF15
sgDDA1
Tasisulam (μM)
d
HeLa
Relative cell viability (%)
sgControl
sgDCAF15
sgDDA1
Tasisulam (μM)
e
ub
ub
RBM39
Tasisulam
E2
DCAF15
RBX1
DDB1
DDA1
CUL4A/B

Fig. S5

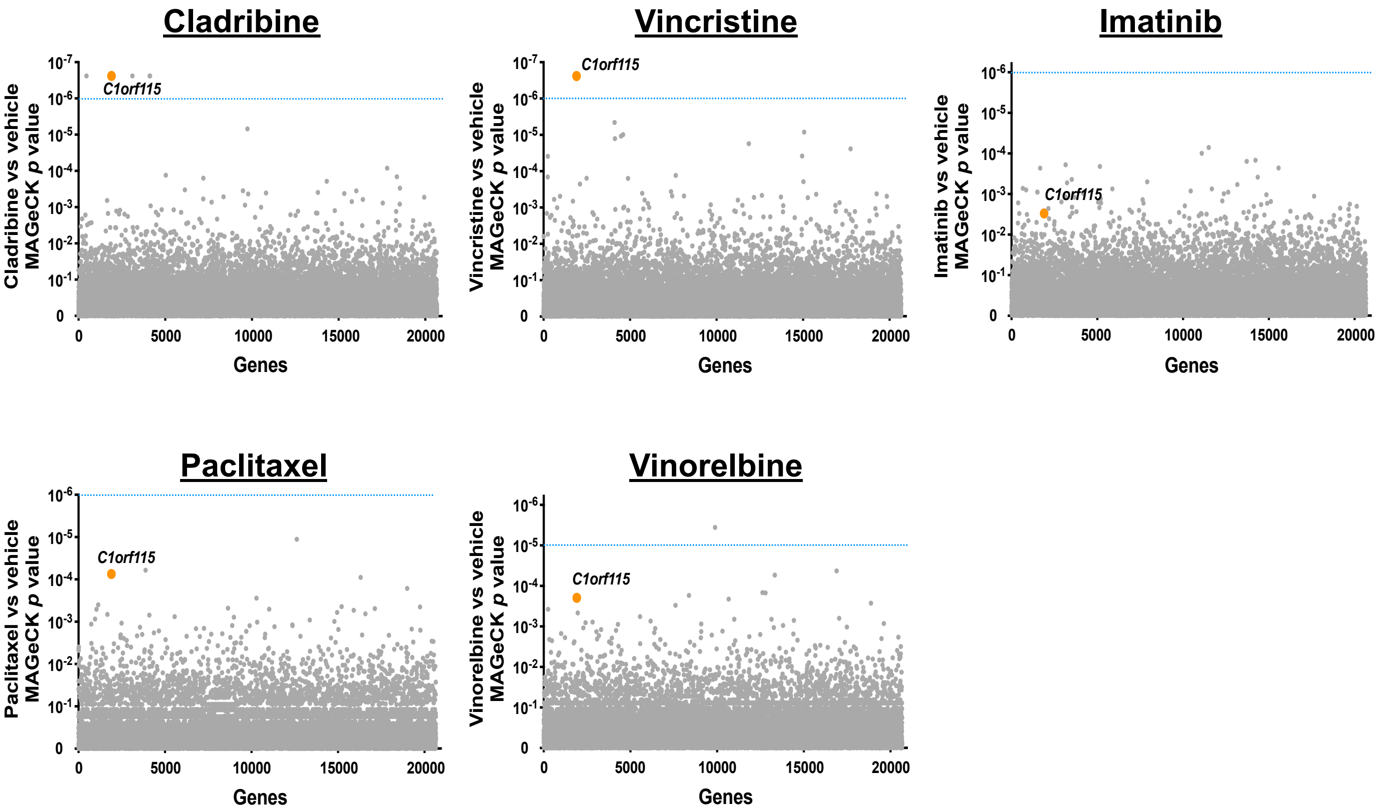
Cladribine
Cladribine vs vehicle MAGeCK p value
C1orf115
Genes
Vincristine
Vincristine vs vehicle MAGeCK p value
C1orf115
Genes
Imatinib
Imatinib vs vehicle MAGeCK p value
C1orf115
Genes
Paclitaxel
Paclitaxel vs vehicle MAGeCK p value
C1orf115
Genes
Vinorelbine
Vinorelbine vs vehicle MAGeCK p value
C1orf115
Genes

Fig. S6

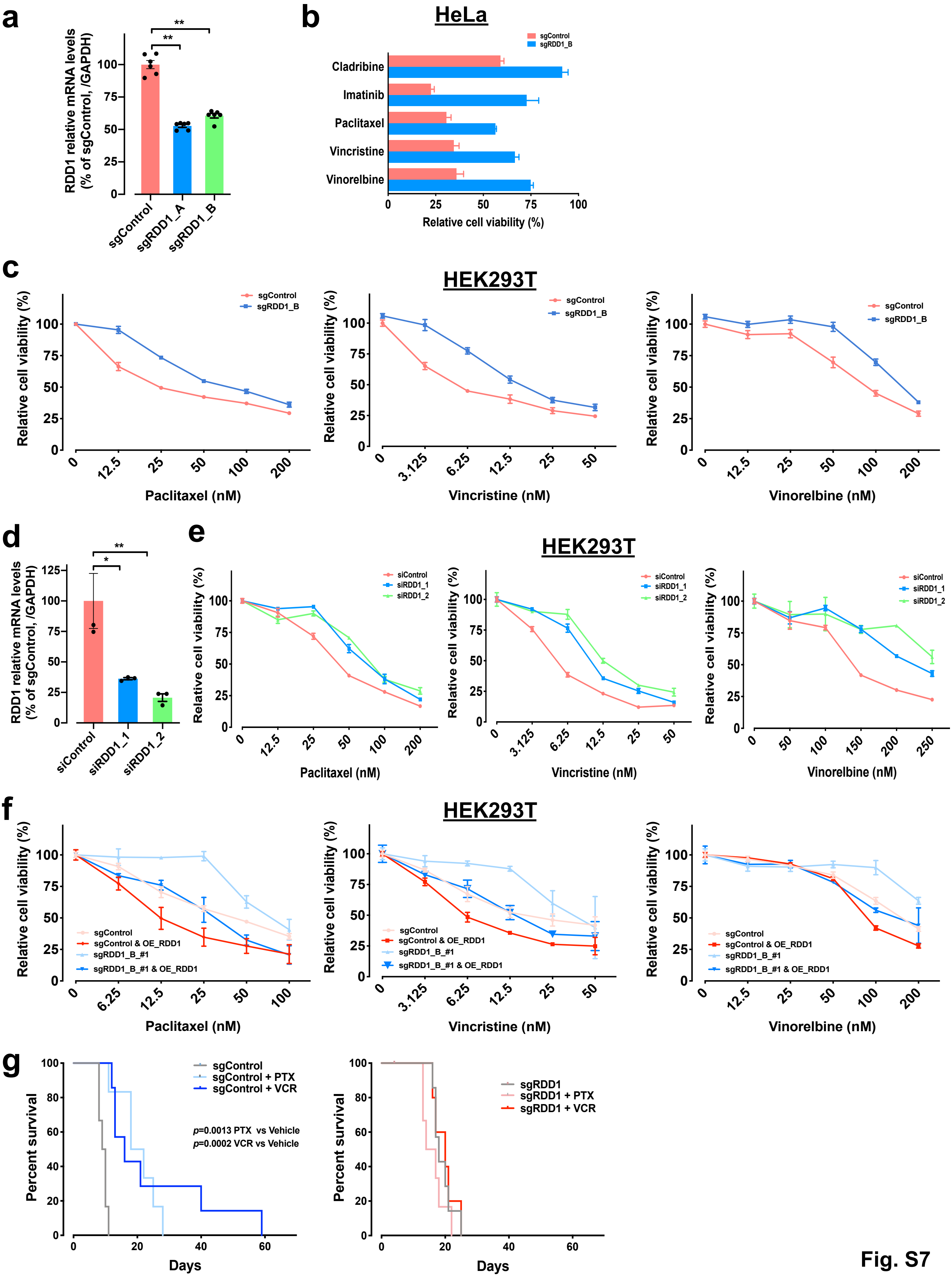
a
RDD1 relative mRNA levels (% of sgControl, /GAPDH)
sgControl
sgRDD1_A
sgRDD1_B
b
HeLa
Cladribine
Imatinib
Paclitaxel
Vincristine
Vinorelbine
Relative cell viability (%)
c
HEK293T
Relative cell viability (%)
Paclitaxel (nM)
Vincristine (nM)
Vinorelbine (nM)
d
RDD1 relative mRNA levels (% of sgControl, /GAPDH)
siControl
siRDD1_1
siRDD1_2
e
HEK293T
f
HEK293T
sgControl & OE_RDD1
sgRDD1_B_#1
sgRDD1_B_#1 & OE_RDD1
g
Percent survival
Days
sgControl + PTX
sgControl + VCR
p=0.0013 PTX vs Vehicle
p=0.0002 VCR vs Vehicle
sgRDD1
sgRDD1 + PTX
sgRDD1 + VCR

Fig. S7

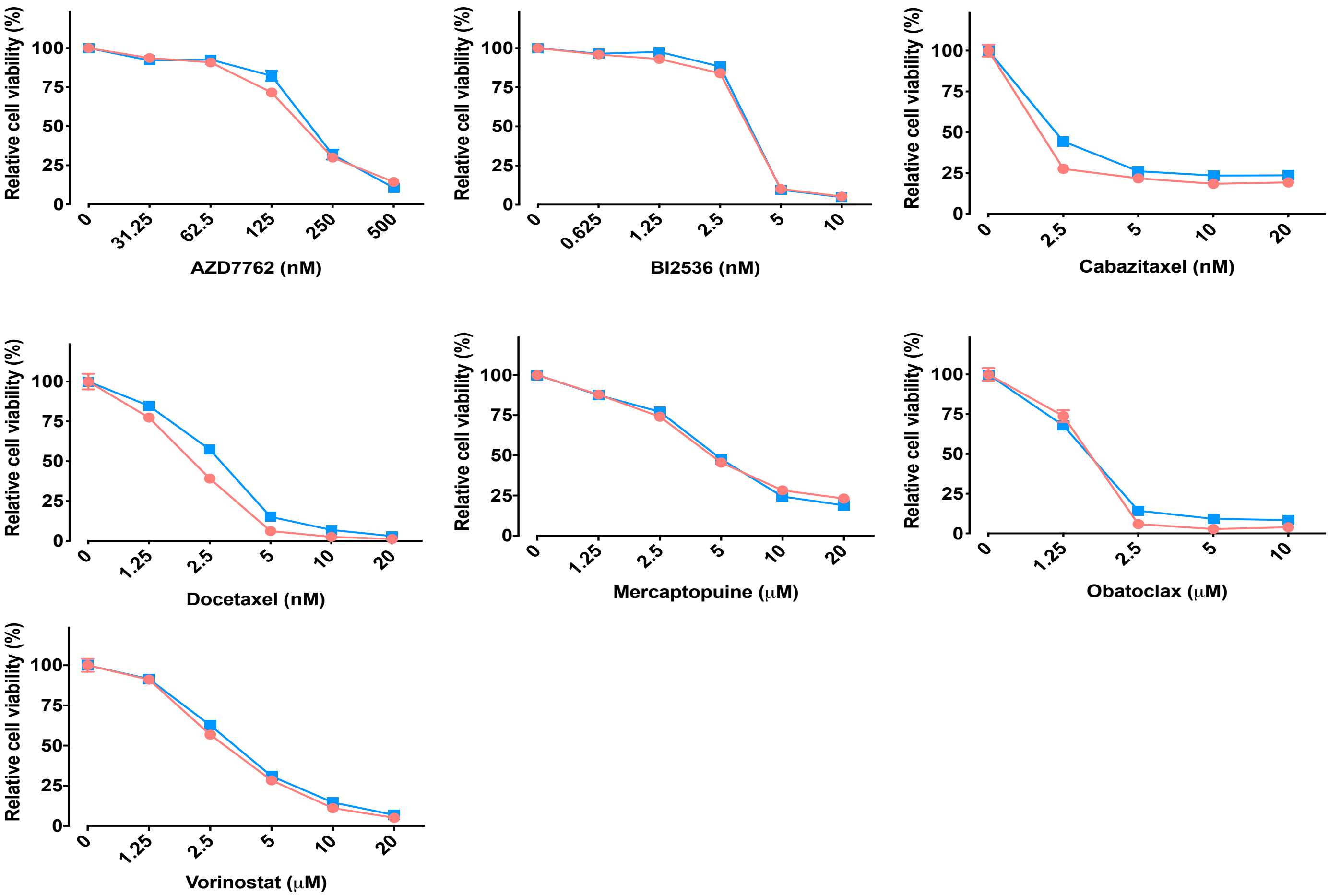
Relative cell viability (%)
AZD7762 (nM)
0
31.25
62.5
125
250
500
BI2536 (nM)
0
0.625
1.25
2.5
5
10
Cabazitaxel (nM)
0
2.5
5
10
20
Docetaxel (nM)
0
1.25
2.5
5
10
20
Mercaptopuine (μM)
Obatoclax (μM)
Vorinostat (μM)
0
25
50
75
100

Fig. S8

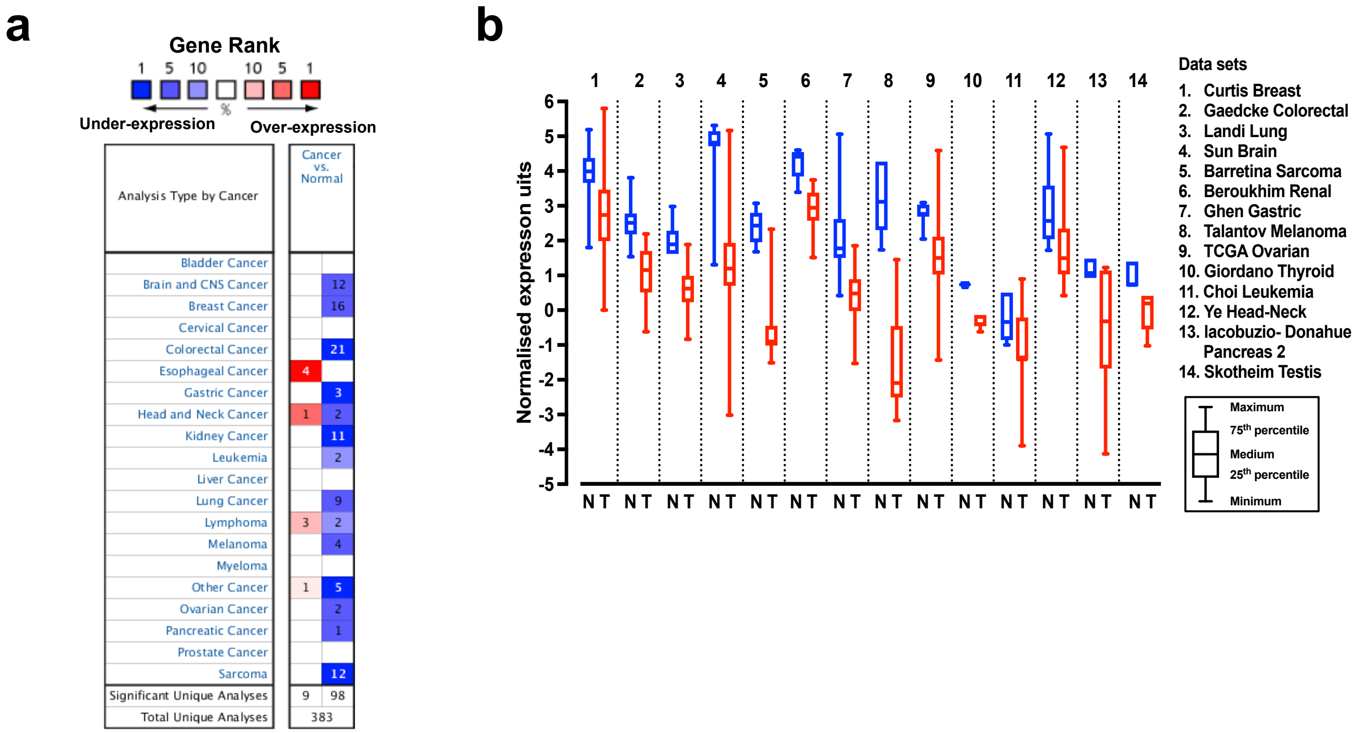

a
Gene Rank
1 5 10 10 5 1
%
Under-expression
Over-expression
Analysis Type by Cancer
Cancer vs. Normal
Bladder Cancer
Brain and CNS Cancer 12
Breast Cancer 16
Cervical Cancer
Colorectal Cancer 21
Esophageal Cancer 4
Gastric Cancer 3
Head and Neck Cancer 1 2
Kidney Cancer 11
Leukemia 2
Liver Cancer
Lung Cancer 9
Lymphoma 3 2
Melanoma 4
Myeloma
Other Cancer 1 5
Ovarian Cancer 2
Pancreatic Cancer 1
Prostate Cancer
Sarcoma 12
Significant Unique Analyses 9 98
Total Unique Analyses 383
b
Normalised expresson uits
N T
Data sets
1. Curtis Breast
2. Gaedcke Colorectal
3. Landi Lung
4. Sun Brain
5. Barretina Sarcoma
6. Beroukhim Renal
7. Ghen Gastric
8. Talantov Melanoma
9. TCGA Ovarian
10. Giordano Thyroid
11. Choi Leukemia
12. Ye Head-Neck
13. Iacobuzio- Donahue Pancreas 2
14. Skotheim Testis
Maximum
75th percentile
Medium
25th percentile
Minimum

Fig. S9

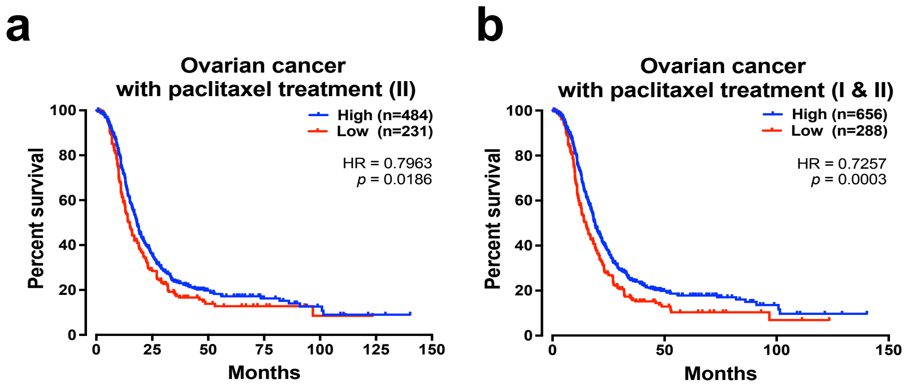
a
Ovarian cancer
with paclitaxel treatment (II)
High (n=484)
Low (n=231)
HR = 0.7963
p = 0.0186
Percent survival
Months
b
Ovarian cancer
with paclitaxel treatment (I & II)
High (n=656)
Low (n=288)
HR = 0.7257
p = 0.0003
Percent survival
Months

Fig. S10

Fig. S1 Drug response curve for all the 27 screened drugs in HAP1 cells
Trypsinized HAP1 cells (7.2×10^4 cells; seeding density 225,000 cells/cm^2) were seeded in each well of a 96-well plate. After 24hrs, various concentrations of anti-cancer drugs were added, and the cells were incubated for an additional 72hrs. Cell viability was analyzed using a resazurin-based assay. The concentration used for screening is indicated by the blue dotted line.

Fig. S2 CRISPR dropout screening identifies HAP1 essential genes
a, Overlap of published HAP1 essential gene sets (pLCV2 and pLCKO) and our HAP1 GeCKO screen.
b, Enrichment pathway analysis of dropout screen hits based on the KEGG database.

Fig. S3 Functional resistance profiles identify known MoA
a, Top enriched genes identified from screens encoding direct therapeutic targets.
b, Schematic of MoA for inhibitors of topoisomerase I and II.
c-f, Enriched genes identified from screens for (c) camptothecin, (d) irinotecan, (e) topotecan and (f) idarubicin.
g, Top enriched genes identified from screens encoding enzyme involved in drug metabolism.
h, Schematic of MoA for nucleoside analogues- cladribine, cytarabine and gemcitabine.
i-k, Enriched genes identified from screens for (i) cytarabine, (j) cladribine and (k) gemcitabine.

Fig. S4 Functional resistance profiles reveal known MoA
a and b, Top enriched genes identified from screens encoding (a) drug transporter, and (b) a downstream mediator of drug action. FDR=0.1 (blue dotted line).
c, Top, enriched genes identified from screens for mercaptopurine (6MP; left) and thioguanine (6TG; right) related to mismatch repair machinery (MMR). FDR=0.1 (blue dotted line). Common enriched genes across two screens within the top 10 (middle), the genes involved in MMR are highlighted in yellow.

Fig. S5 Loss of DCAF15 or DDA1 leads to tasisulam resistance
a, Chemical structure of tasisulam.
b, Enriched genes identified from screens for tasisulam. FDR=0.1 (blue dotted line).
c and d, CRISPR-Cas9 targeted cells decreased their sensitivity to tasisulam in (c) HAP1 and (d) HeLa cells.
e, Schematic of the drug MoA for tasisulam.

Fig. S6 Identification of novel multi-drug resistance gene *C1orf115*/ *RDD1*
C1orf115/ *RDD1* gene identified from screens including cladribine, imatinib, paclitaxel, tasisulam, vincristine and vinorelbine. FDR=0.1 (blue dotted line).

Fig. S7 Loss of RDD1 results in multiple drug resistance

a, *RDD1* mRNA levels were analyzed by RT-qPCR in HAP1 RDD1 KO cells. All data represented as mean ± S.E.M (n=6). One-way ANOVA followed by Tukey's *post hoc* test, **, p <0.01.

b and c, CRISPR-Cas9 targeted cells decreased their sensitivity to drugs in (c) HeLa and (d) HEK293T cells.

d, *RDD1* mRNA levels were analyzed by RT-qPCR in siRNA targeting RDD1 in HAP1 cells. All data represented as mean ± S.E.M (n=3). One-way ANOVA followed by Tukey's *post hoc* test, **, p <0.01.

e, siRNA targeting RDD1 cells decreased their sensitivity to drugs in HEK293T cells.

f, Overexpression of sgRNA resistant RDD1 (OE_RDD1) rescues the loss of RDD1-induced drug resistance in HEK293T cells. HEK293T control cells (sgControl) and clonal CRISPR-Cas9 targeted cells (sgRDD1_B_#1) were transiently transfected with pLenti-C-mGFP and gRNA-resistant constructs (OE_RDD1) for 72hrs and subjected to drug treatments and cell viability assays.

g, CRISPR-Cas9 *RDD1* targeting HAP1 cells decreased their sensitivity to vincristine (VCR) and paclitaxel (PTX) in xenograft tumor model. Overall survival is shown. All data represented as mean ± S.E.M (n = 4-6 mice per group). *P* values were calculated using the log-rank (Mantel-Cox) test.

Fig. S8 CRISPR-Cas9 targeted RDD1 cells do not display broad resistance in HeLa cells

Fig. S9 RDD1 expression in human cancers

a, RDD1 expression in different cancers using oncomine database (www.oncomine.org). Cell color is determined by the best gene rank percentile for the analyses within the cell. NOTE: An analysis may be counted in more than once cancer type.

b, Oncomine database analysis of gene expression of RDD1 are significantly downregulated in different cancers (T) relative to normal tissue (N). Only the most significant representative data set in the particular cancers are shown.

Fig. S10 Low *RDD1* expression is significantly associated with poor patient survival a and b, Kaplan-Meier survival plots of patient overall survival in ovarian cancer with paclitaxel treatment in (a) second patient cohort and (b) combined cohorts using the Kaplan-Meier Plotter.
