# Supplementary material for: Systematic functional identification of cancer multi-drug resistance genes
Source: Genome Biol. 2020 Feb 7;21:27. doi: 10.1186/s13059-020-1940-8 (PMC7006212; doi:10.1186/s13059-020-1940-8)
Supplement: Supplementary file 6 — Additional file 6. Review history. [file 13059_2020_1940_MOESM6_ESM.docx]

Review History

**First round of review**

**Reviewer 1**

**Are you able to assess all statistics in the manuscript, including the appropriateness of statistical tests used?**

Yes, and I have assessed the statistics in my report.

**Comments to author:**

Overall summary of paper

The authors describe a genome-wide CRISPR knockout screen in a near-haploid cell line for modifiers of resistance to 27 anti-cancer agents. They identify a number of genes for each compound that are either novel or have previously been implicated in resistance.

A major concern is that it is not clear what cut-off the authors use to define significant resistance genes from the MAGeCK analysis. The majority of studies based on MAGeCK analyses of CRISPR data use FDR values as a cut-off (e.g. 0.25 or 0.1) rather than the p-values given the risk of the latter with multiple hypothesis testing (and for that reason there are often 100s - 1000s of 'significant' genes using p-value). Use of FDR in this paper would disqualify a large number of the genes that the authors highlight to support the validity of the screen but is in my opinion essential for a more robust analysis.

Additionally, resistance experiments in particular are critically dependent on careful selection of the drug concentration - this needs to be discussed and defined for each compound.

In the latter sections of the paper there is a focus on a novel resistance gene, C1orf115. It is not clear to me why this was selected from the larger set of recurrent resistance genes.


Specific comments

1.     Why use HAP1 cell line? Numerous studies have shown that CRISPR/Cas9 libraries are able to efficiently silence genes even in diploid or aneuploid cells (such as cancer cell lines), which arguably are more clinically relevant that a near-haploid cell line from a chronic myeloid leukaemia cell line.

2.     All figures that include the MAGeCK p-value should indicate using a dotted line FDR values e.g. 0.25 or 0.1. to allow reader to assess likelihood of events being false positives.

3.     For resistance screens, it is important to use a concentration of drug which will exert a sufficiently strong positive selective pressure on those cancer cells harbouring sgRNA for resistance genes. This is generally achieved by treating with greater than IC50-60 concentration of drug. Page 3, Line 42 - what was the minimum lethal concentration of drug used in these cells expressed as IC value? What does the Maximum screening concentration listed in Table S1 refer to? From review of Table S3, many of the compounds have only a small number of hits at FDR<0.25 - this is often because the selective pressure (drug concentration) is either too low or too high.

4.     MAGeCK analysis - versions 0.5.6 and higher provide a log FC value - this should be included in Table S3 to show the magnitude of the resistance effect.

5.     Figure 1f - I would remove the left-sided panel of drugs - it's slightly confusing and is unnecessary given the drugs are already colour-coded for class. I'd add a legend for what the different colours represent.

6.     Page 3, Line 46-48 - what significance FDR threshold was used?

7.     Page 4, Line 53 - Figure 2f does not display data for Olaparib - in Table S3, olaparib - PAPR1 and PARP2 have FDR of 0.8 and 0.93 - these are not significant.

8.     Figure 2a - the p-value and positive score from MAGeCK are highly related and this plot therefore doesn't tell me very much - more informative (and more typical for CRISPR screen papers) is a plot of -log10 p-value or FDR versus the lfc (log FC) value for each drug/gene.

9.     Page 5, line 40+ - many of these resistance genes are not significant (even at a relatively non-stringent FDR cut-off of 0.25 - these should be excluded or an explanation given as to why they are considered relevant to this paper.

10.     PAC-1 section - ELAVL1 is not a significant hit in the screen (FDR 0.918) - why then does it confer resistance and why was it selected for discussion? What concentration of PAC-1 was used in the CRISPR screen compared to the concentration range in Fig 3e? In fact, no genes were identified as being under positive selection using FDR cut-off criteria.

11.     Page 7, line 33 - what significance threshold was used? From methods it would appear that a cut-off of FDR <0.1 or highly ranked (top 100, unadjusted p-value <0.01) was used? What is the justification for using the highly ranked method - this is not a robust use of these statistical measures and will be prone to false positive hits.

12.     Page 7, line 46+ - analysis of TCGA data for these MDR genes was based upon freq of non-silent mutations - this instead should be putative loss-of-function mutations (ie. frameshift indels, essential splice and nonsense) or copy number loss - given the resistance effects from the CRISPR screens are via loss of function of these genes.

13.     C1orf 115 (RDD1) - this gene was only detected as a significant resistance event (FDR<0.25) in 2 drugs (Cladarabine, Vincristine) - why was this selected for further investigation over other recurrent hits? In Paclitaxel, FDR 0.46 and Vinorelbine 0.54.

14.     RDD1 characterisation - I can't see any WB showing loss of expression with sgRDD1_A and _B - these should be included - preferably in Figure 6d.

**Reviewer 2**

**Are you able to assess all statistics in the manuscript, including the appropriateness of statistical tests used?**

Yes, and I have assessed the statistics in my report.

**Comments to author:**

Summary:
In order to elucidate genetic factors that contribute to drug sensitivity and resistance, the authors perform a series of pooled genome-wide CRISPR-Cas9 screens for 27 different anti-cancer drugs in HAP1 cells using the genome-scale GeCKOv2 library from the Broad Institute. After a 14-day treatment at a minimal lethal concentration for each of the drugs, cells were harvested and shRNA abundance was quantified relative to a control population using MAGeCK to score significantly enriched hits. Analysis of cellular distribution of the resistance targets indicated the contribution of multiple cellular compartments to drug MoA or resistance. Hierarchical cluster analysis was then used to classify the 27 compounds based on similarity of resistance gene overlap from functional resistance profiles. The authors state that "in general, classical drug classifications translated to similar overall resistance gene sets, however in some instances genetic resistance deviated from expected profiles". Notably, drugs targeting topo I and topo II showed vastly dissimilar molecular resistance profiles, as did PAC-1 and tasisulam, which are drugs that activate caspases. Importantly, functional resistance profiles revealed known MoA (e.g. TOP1 for camptothecin, irinotecan, topotecan; TOP2A for idarubicin; PARP1 and PARP2 for olaparib; DCK for cladribine, cytarabine and gemcitabine; DHFR and SLC19A1  for methotrexate; CDC25A for AZD7762; MLH1, MSH2 and MSH6 for mercaptopurine and thioguanine).

Interestingly, functional resistance profiles also revealed novel modes of drug action (e.g. AK2- cladribine; C1orf115 - vincristine; FGF12 - RO3066; FAM180A - obatoclax; KDM1A - vinorelbine; MKNK2 - imatinib; NSD1 - paclitaxel; OSTC - PAC-1; PPP6C - docetaxel; SLC43A2 - oxaliplatin; ZNF774 - sorafenib; NAA60 - methotrexate; etc..). Because the authors profiled 27 drugs, they also found genes that mediate resistance to more than one anti-cancer drug (e.g. YBX1 - AZD7762 and cisplatin).

The authors then describe a total of 41 multi-drug resistance genes (MDR), significantly enriched with at least 3 or more drugs and at least 2 independent sgRNAs per genes (i.e. core MDR genes). These were then classified by their cellular roles, including pro-apoptotic genes (e.g. BAX, BID, FADD, PMAIP1/NOXA), chromatin organisation (NSD1, SMARCB1), a putative DNA/RNA helicase (SLFN11), deoxycytidine kinase (DCK), and topoisomerase I (TOP1). Several of these genes were frequently mutated in human cancers. Overall, the authors identify 32 novel putative MDR genes, including the uncharacterized gene C1orf115, which they rename RDD1 (Required for Drug-induced Death 1).

RDD1 encodes a small ~14kDa protein with a DUF4710 domain of unknown function that is highly conserved in vertebrates (mammals, marsupials and birds). RDD1 is broadly expressed with highest expression across the gastrointestinal tract, brain and female reproductive system. Loss-of-function of RDD1 resulted in resistance to at least 5 anti-cancer drugs (e.g. paclitaxel, vincristine, vinorelbine). However, RDD1 did not display broad resistance. Notably, RDD1 loss conferred resistance to vincristine in HELA tumors xenografted into mice. And RDD1 mRNA expression is correlated with poor patient survival in multiple different cancers. These observations suggest that RDD1 could be a valuable biomarker for outcome and prediction of drug resistance.

To summarize, the methods are appropriate for the aims of the study, they are well described, and controls have been properly included. The conclusions are adequately supported by the data; however, see comment 1 below. Additional methods details would be helpful for data replication (e.g. drug names and doses). The work represents a significant advance as there is no systematic chemical genetic analysis of drug resistance mechanisms published using genome-scale pooled CRISPR screening. And the paper would be of broad interest, particularly if all of the primary data is published for re-analysis.

Overall, I was enthusiastic about the idea of building chemo-genetic profiles for human cancer cells. Despite some of the presentation issues in this manuscript (i.e. figures), there is much potential to utilize this information to deepen our knowledge of chemical genetic interactions in human cells.

Comments:
1. The figures were all low resolution, which created a lot of guess work for this reviewer. For example, it was impossible to read any of the drug names in figure 1. Figure 4 was completely illegible. A higher resolution version of the figures would have made a big difference. Overall, it was difficult to assess many of the figure panels.

2. Cross-resistance: This term was confusing and could be clarified for better read-ability. It would make more sense to call them "core drug resistance genes", "common drug resistance genes", or possibly "multi-drug resistance genes". Cross-resistance needs to be clearly defined or better terminology used.

3. What was the "minimal lethal concentration" for each anti-cancer agent? What was the drug dosing schedule for each of the drugs? Full details of all drug doses and scheduling should be included so that the data can be reproduced.

4. How did essential genes drop out in each of the drug screens? It wasn't clear how this data was normalized.

5. How did the authors account for sgRNA variability in the MaGECK analyses? In other words, how did each of the sgRNAs perform on a per gene basis? Could the authors clarify whether the multi-drug resistance phenotype of RDD1 and other genes was driven by multiple guides and not single outlier guides, which can sometime enrich in positive selection screens because they are highly represented in the starting pool.

6. There are many genome-wide pooled CRISPR screens now published. Are the drug-resistance genes presented in this manuscript related to any gene co-dependency clusters in the DepMap (www.depmap.org) data, for example?

7. Does overexpression of RDD1 cause increased drug sensitivity? A rescue experiment would be helpful to convince readers that RDD1 itself is necessary for the effect, and not something about the RDD1 locus. For example, expression of RDD1 in RDD1 knockout cells would provide good evidence that the CRISPR effect is specific to the RDD1 gene.

8. How many cell lines have the authors tested where loss of RDD1 leads to resistance to drug-induced toxicity? A broader panel of cell lines would help to define the contribution of RDD1 to genotype-specific resistance to drug-induced toxicity.

9. The authors do not provide any data on the molecular function of RDD1, but name the protein. It would be valuable to speculate on the function of the protein, its localization, interaction partners, etc…

**Authors Response**

**Point-by-point responses to the reviewers’ comments:**

Reviewer #1: Overall summary of paper

The authors describe a genome-wide CRISPR knockout screen in a near-haploid cell line for modifiers of resistance to 27 anti-cancer agents. They identify a number of genes for each compound that are either novel or have previously been implicated in resistance.

1. A major concern is that it is not clear what cut-off the authors use to define significant resistance genes from the MAGeCK analysis.
The majority of studies based on MAGeCK analyses of CRISPR data use FDR values as a cut-off (e.g. 0.25 or 0.1) rather than the p-values given the risk of the latter with multiple hypothesis testing (and for that reason there are often 100s - 1000s of 'significant' genes using p-value).
Use of FDR in this paper would disqualify a large number of the genes that the authors highlight to support the validity of the screen but is in my opinion essential for a more robust analysis.

*Response: We agree with the reviewer this is an important issue. While using a highly stringent FDR can help us avoid false positives, we find that only relying on a stringent FDR threshold to assess these data results in biologically meaningful resistance genes (e.g. PAC1-ELAVL1, AZD7762-YBX1) being excluded.

To address this concern, we have now re-analysed our data, and have employed different levels of stringency. This analysis is included in the revised manuscript (see page 13; line 10-22) and is summarised here:

- Tier 1 (most stringency) hits must have an FDR<0.25, at least two supporting sgRNAs (“pos.good.sgrna” in Table S3) and an unadjusted of p<0.01 (Table S3).
- Tier 2 (moderate stringency) hits must have at least two supporting sgRNAs and an unadjusted p<0.01 (Table S3).
- Tier 3 (minimal stringency) hits must have unadjusted p<0.01 (Table S3);

This new tiered analysis is presented in new Fig S3a and method (see page 13; line 10-22). Moreover, when we mention resistance genes in the text the tier level is provided. Based on our screening of multiple drugs with related mechanisms of actions (such as topo I inhibitors; see Fig. S2d, Fig S2E gemcitabine and cytarabine) we find tier 3 hits can reproducibly show resistance and are not strictly false positive signals. However, we acknowledge that there is an increased likelihood of events being false positives using only p<0.01 cut-off (Tier 3 hits), and clarified this in the revised manuscript (see page 4; line 14-19).

Since the primary goal of this study was to identify multi-drug resistance (MDR) genes, we have further included an additional level of stringency in the MDR analysis. Candidate MDR genes were selected from tier 1 or 2 in the primary screens (two supporting sgRNAs and an unadjusted p<0.01), and hits must show resistance to at least 3 drugs. Then from this set, the MDR FDR was calculated and an MDR FDR <0.05 was considered significant (Table S7). This new analysis is described in the methods (see page 14; Explicit testing for multi-drug resistant (MDR) genes section). We hope the reviewer will accept this new much more stringent analysis.*

2. Additionally, resistance experiments in particular are critically dependent on careful selection of the drug concentration - this needs to be discussed and defined for each compound.

& 6. For resistance screens, it is important to use a concentration of drug which will exert a sufficiently strong positive selective pressure on those cancer cells harbouring sgRNA for resistance genes. This is generally achieved by treating with greater than IC50-60 concentration of drug. Page 3, Line 42 - what was the minimum lethal concentration of drug used in these cells expressed as IC value? What does the Maximum screening concentration listed in Table S1 refer to? From review of Table S3, many of the compounds have only a small number of hits at FDR<0.25 - this is often because the selective pressure (drug concentration) is either too low or too high.

*Response: Our 14-day protocol involved an initial 3 days of high concentration drug resistance screening (at IC90-99) followed by lower selection pressure on the resistant cells over the remaining 11 days. To highlight this change in dose, we described the initial dose as “maximum screening concentration” however we understand this is confusing. To resolve this issue, we have replaced “maximum screening concentration” with “IC90-99” (Table S1). For each drug, the IC90-99 was calculated from a 3 day-dose response experiments and these data plus IC90-99 values are presented in new figure S1 and we have also clarified this further in the text (page 3 line 45-50). Moreover, the reviewer is correct, due to the variable efficacies and actions for the multiple drugs compared, in some instances we have likely used a stronger selective pressure and this could result in less significant hits at FDR<0.25.*

3. In the latter sections of the paper there is a focus on a novel resistance gene, C1orf115. It is not clear to me why this was selected from the larger set of recurrent resistance genes.

& 16. C1orf 115 (RDD1) - this gene was only detected as a significant resistance event (FDR<0.25) in 2 drugs (Cladarabine, Vincristine) - why was this selected for further investigation over other recurrent hits? In Paclitaxel, FDR 0.46 and Vinorelbine 0.54.

*Response: In our initial analysis of these screening data, we used a rank model similar to that of the original CRISPR screening papers [1]. From this, C1orf115/RDD1 was consistently highly ranked across 5 drugs. We then functionally validated this result with independent guides and cell lines in vitro and RDD1 KO lines also showed drug resistance in vivo (See new Fig 6, and Fig. S10). However, as the field of screening has since advanced we have altered our statistical analysis accordingly. Importantly, our validation data supports that RDD1 is a bonafide MDR gene, and this is something we would have missed had we only used an FDR<0.05 cut off for these analysis. Moreover, during revision we have refined our analysis further, especially with respect to comparisons across multiple screens, and our new analysis highlights that C1orf115/RDD1 shows MDR significance at FDR<0.05 for 5 drugs (new Table S7), and this is the highest ranked novel multidrug resistance gene identified in this study.*

4. Why use HAP1 cell line? Numerous studies have shown that CRISPR/Cas9 libraries are able to efficiently silence genes even in diploid or aneuploid cells (such as cancer cell lines), which arguably are more clinically relevant that a near-haploid cell line from a chronic myeloid leukaemia cell line.

*Response: HAP1 cells have been commonly used for different functional genetic screens [2-6] and we wanted to compare functional resistance profiles within this well characterised cell line. Moreover, this cell line has a haploid genome which can augment the sensitivity of CRISPR loss-of-function screening. In this regard, our resistance profiles were able to identify a number of well-known resistance genes for many drugs tested, and this highlights the utility of screening HAP1 cells. Finally for many resistance hits, we have validated these activities in other cell lines (Hela and also now HEK293).*

5. All figures that include the MAGeCK p-value should indicate using a dotted line FDR values e.g. 0.25 or 0.1. to allow reader to assess likelihood of events being false positives.

*Response: As suggested by reviewer, we added a dotted line indicating the FDR=0.25 in all MAGeCK p-value figures.*

7. MAGeCK analysis - versions 0.5.6 and higher provide a log FC value - this should be included in Table S3 to show the magnitude of the resistance effect.

*Response: We added the log FC value for each drug in new Table S3, and sgRNA level data in new Table S2 to provide more detail information of the enrichment or depletion of sgRNA targeting the gene in each screen.*

8. Figure 1f - I would remove the left-sided panel of drugs - it's slightly confusing and is unnecessary given the drugs are already colour-coded for class. I'd add a legend for what the different colours represent.

*Response: As suggested by reviewer, we remove the old Fig.1f to avoid confusing since the clustering information is given in New Fig. 1f. The colors used in Figure 1f match the classification used in Figure 1a.*

9. Page 3, Line 46-48 - what significance FDR threshold was used?

*Response: Please see our above response to comment #1. We have now provided the tier level for each hit in the revised manuscript (see page 4; line 14-19).*

10. Page 4, Line 53 - Figure 2f does not display data for Olaparib - in Table S3, olaparib - PAPR1 and PARP2 have FDR of 0.8 and 0.93 - these are not significant.

*Response: We appreciate the reviewers concern and we have removed this figure.*

11. Figure 2a - the p-value and positive score from MAGeCK are highly related and this plot therefore doesn't tell me very much - more informative (and more typical for CRISPR screen papers) is a plot of -log10 p-value or FDR versus the lfc (log FC) value for each drug/gene.

*Response: As suggested by reviewer, we revised Fig.2A using -log10(positive score) vs logFC(top 2 sgRNAs) for each drug/gene.*

12. Page 5, line 40+ - many of these resistance genes are not significant (even at a relatively non-stringent FDR cut-off of 0.25 - these should be excluded or an explanation given as to why they are considered relevant to this paper.

*Response: We appreciate the reviewers concerns. In the revised manuscript we have provided the tier level for these resistance genes in the revised manuscript (Page 5, Line 20+).*

13. PAC-1 section - ELAVL1 is not a significant hit in the screen (FDR 0.918) - why then does it confer resistance and why was it selected for discussion? What concentration of PAC-1 was used in the CRISPR screen compared to the concentration range in Fig 3e? In fact, no genes were identified as being under positive selection using FDR cut-off criteria.

*Response: We found that ELAVL1 are supported by all the 6 sgRNAs (gRNA FDR<0.001; Table S2) targeting this gene (Fig. 3c). And we have validated this gene that is a biological hit (Fig. 3) indicating that it is a false negative if using only FDR cut-off criteria.*

14. Page 7, line 33 - what significance threshold was used? From methods it would appear that a cut-off of FDR <0.1 or highly ranked (top 100, unadjusted p-value <0.01) was used? What is the justification for using the highly ranked method - this is not a robust use of these statistical measures and will be prone to false positive hits.

*Response: To address the reviewer’s concerns and reduce the risk of false positive hits, we reanalysed our data using more stringent testing method (see Method section-“Explicit testing for multidrug resistant (MDR) genes”), resulting in 19 MDR genes (“MDR FDR”<0.05; Table S7).*

15. Page 7, line 46+ - analysis of TCGA data for these MDR genes was based upon freq of non-silent mutations - this instead should be putative loss-of-function mutations (ie. frameshift indels, essential splice and nonsense) or copy number loss - given the resistance effects from the CRISPR screens are via loss of function of these genes.

*Response: As suggested by reviewer, we have reanalysed the data showing the loss-of-function mutations and copy number loss for the MDR genes (Fig. 5b).*

17. RDD1 characterisation - I can't see any WB showing loss of expression with sgRDD1_A and _B - these should be included - preferably in Figure 6d.

*Response: Western blotting RDD1 has proven to be difficult. We first tried to blot RDD1 using a commercially available antibody (from Sigma) however this was not successful using a variety of conditions. We next attempted to generate a new RDD1 antibody. We first tried expressing full length recombinant RDD1 in an attempt to generate a rabbit polyclonal antibody, however we were unsuccessful. We next generated a truncated Fc-tagged RDD1 (residues 2-115 of the N terminus) and used phage display to isolate interacting clones, and interactions were then confirmed using BioLayer Interferometry. We then tested 7 biotinylated phage fragment (scFv-Fc) “antibodies” that showed specificity by BioLayer Interferometry however again could not reliably detect endogenous RDD1. We then performed cellular localisation of RDD1 and found RDD1 reliably localised to the subdomains of the nucleus which could be euchromatic (since they exclude Hoechst) or the nucleolus which can also exclude Hoechst (see Fig. 6h. Based on these observations, we hypothesised that RDD1 western blotting was difficult because RDD1 was precipitating with the insoluble fraction during cell lysis. To address this, cells were lysed with high salt RIPA, sonicated to solubilised RDD1, and then subjected to western blotting. This proved successful, and we could visualise RDD1 by western blotting with the commercial Sigma antibody (see Fig. 6c and i). Using this protocol, we observed RDD1 targeting resulting in 25-45% reduction of RDD1. Based on the cellular localisation and western blotting results, we next fractioned cells using a commercial kit (Sigma) and found RDD1 concentrated in the insoluble nuclear fraction which contains both chromatin and the nucleolus (see Fig. 6i). We feel these new data improve the quality of the manuscript and thanks the reviewer for this suggestion.*

Reviewer #2:

1.The figures were all low resolution, which created a lot of guess work for this reviewer. For example, it was impossible to read any of the drug names in figure 1. Figure 4 was completely illegible. A higher resolution version of the figures would have made a big difference. Overall, it was difficult to assess many of the figure panels.

*Response: We apologise for this, our figures were high resolution TIF when we uploaded, however conversion to PDF made the figures blurry. It seems to be the result of the manuscript submission system. However, in the final PDF version, there is a link at the top right of each figure which allows you to download the high quality TIF version. In order to resolve this issue, we now upload our figures in PDF format. We hope that this is acceptable solution.*

2. Cross-resistance: This term was confusing and could be clarified for better read-ability. It would make more sense to call them "core drug resistance genes", "common drug resistance genes", or possibly "multi-drug resistance genes". Cross-resistance needs to be clearly defined or better terminology used.

*Response: We agree and have revised in the main text.*

3. What was the "minimal lethal concentration" for each anti-cancer agent? What was the drug dosing schedule for each of the drugs? Full details of all drug doses and scheduling should be included so that the data can be reproduced.

*Response: We now provide dose-response curve of drugs studied in Fig. S1. We used the kill curve of each drug to determine the drug concentration in order to achieve the “minimal lethal concentration” (IC90-99) in each screen.

The “Maximum screening concentration” (Table S1) that we refered to the initial concentration (IC90-99) using for screening. To resolve this issue, we have replaced “maximum screening concentration” with “IC90-99” (Table S1). In the screening, initial drug concentrations for selection were highest (IC90-99) and then over the course of continued selection the drug concentrations were then lowered to minimum levels required to continue killing non-transduced wild-type cells. We have now clarified this in the revised manuscript (see page 3 line 45-50).*

4. How did essential genes drop out in each of the drug screens? It wasn't clear how this data was normalized.
& 5. How did the authors account for sgRNA variability in the MaGECK analyses? In other words, how did each of the sgRNAs perform on a per gene basis? Could the authors clarify whether the multi-drug resistance phenotype of RDD1 and other genes was driven by multiple guides and not single outlier guides, which can sometime enrich in positive selection screens because they are highly represented in the starting pool.

*Response: As suggested by reviewer, we have compared our dropout screen results to a published genome-scale CRISPR dropout screen that was performed in HAP1 cells [7]. Our data show substantial overlap between these three sets of essential genes (Fig. S2a) and most of the enrich pathways from our essential gene set are involved in critical biological functions (Fig. S2b and Table S4). Also, we have added the sgRNA level CRISPR screen data for each screen in new Table S2 to provide more detail information of the enrichment or depletion of sgRNA targeting the gene in each screen. All the genes we validated and the MDR genes (Table S7) are supported by multiple gRNAs from screens (At least Tier 2 hits, see Method section; page 13; line 10-22). As shown in Fig. S10a, for RDD1, we clearly see there is no single sgRNA driving the effect, but multiple guides across many drugs.*

6. There are many genome-wide pooled CRISPR screens now published. Are the drug-resistance genes presented in this manuscript related to any gene co-dependency clusters in the DepMap (www.depmap.org) data, for example?

*Response: We performed gene co-dependency analysis on the 19 MDR genes using the DepMap and these results are shown in new Table S7.*

7. Does overexpression of RDD1 cause increased drug sensitivity? A rescue experiment would be helpful to convince readers that RDD1 itself is necessary for the effect, and not something about the RDD1 locus. For example, expression of RDD1 in RDD1 knockout cells would provide good evidence that the CRISPR effect is specific to the RDD1 gene.

*Response: As suggested by reviewer, we performed the rescue experiment to show that RDD1 itself is necessary for the effect, and not something about the RDD1 locus (in Fig. S10d). Also, overexpression of RDD1 in HEK293T sgControl cells results in more sensitivity to the drugs (please see Fig. S9d).*

8. How many cell lines have the authors tested where loss of RDD1 leads to resistance to drug-induced toxicity? A broader panel of cell lines would help to define the contribution of RDD1 to genotype-specific resistance to drug-induced toxicity.

*Response: To test if the observed resistance is generalisable, we tested in HAP1 (Fig. 6), HeLa and HEK293 cells (in Fig. S10b and c).*

9. The authors do not provide any data on the molecular function of RDD1, but name the protein. It would be valuable to speculate on the function of the protein, its localization, interaction partners, etc…

*Response: To determine the localisation of RDD1, we tagged the C-terminus with GFP and performed confocal microscopy in HEK293T and HeLa cells. Here, we find the signal primarily restricted to the nucleus and particularly concentrated in the nucleolus. The RDD1-GFP signal throughout the nucleus fluoresces in a distinct space to the Hoechst signal, which binds to heterochromatin but can be excluded from euchromatin and the nucleolus (Fig. 6h). The highly concentrated nucleolar RDD1-GFP signal resembles the fluorescence pattern of proteins expressed in the granular component of the nucleolus such as B23 [8]. By colocalisation, we found the RDD1-GFP signal appears to be nucleolar but excluded from the dense fibrillar component/fibrillar centre which we visualise by staining with fibrillarin, a marker for this compartment (Fig. 6h). Importantly, RDD1-GFP fusion protein can rescue drug resistance phenotypes when transiently expressed in RDD1 KO HEK293T cells (Fig. S10d), suggesting that the fusion protein functions properly within the cell.

In parallel we tried to optimise detection of endogenous RDD1 by western blotting, which was difficult. We first tried to blot RDD1 using a commercially available antibody (from Sigma) however this was not successful using a variety of conditions. We next attempted to generate a new RDD1 antibody. We first tried expressing full length recombinant RDD1 in an attempt to generate a rabbit polyclonal antibody, however we were unsuccessful. We next generated a truncated Fc-tagged RDD1 (residues 2-115 of the N terminus) and used phage display to isolate interacting clones, and interactions were then confirmed using BioLayer Interferometry. We then tested 7 biotinylated phage fragment (scFv-Fc) “antibodies” that showed specificity by BioLayer Interferometry for use with western blot however again could not reliably detect endogenous RDD1. Based on our RDD-GFP imaging studies, we hypothesised that RDD1 western blotting was difficult because RDD1 was precipitating with the insoluble nucleolar fraction during cell lysis. To address this, cells were lysed with high salt RIPA, sonicated to solubilised RDD1, and then subjected to western blotting. This proved successful, and we could visualise RDD1 by western blotting with the commercial Sigma antibody (see Fig. 6c and 6i). Based on the cellular localisation and western blotting results, we next fractioned cells using a commercial kit (Sigma) and found RDD1 concentrated in the insoluble nuclear fraction which contains both chromatin and the nucleolus (see Fig. 6i). Thus, our data supports the notion that RDD1 is a novel nucleolar factor. We feel these new data improve the quality of the manuscript and thanks the reviewer for this suggestion.

Reference:
1. Shalem O, Sanjana NE, Hartenian E, Shi X, Scott DA, Mikkelsen TS, Heckl D, Ebert BL, Root DE, Doench JG, Zhang F: Genome-scale CRISPR-Cas9 knockout screening in human cells. Science 2014, 343:84-87.
2. Carette JE, Raaben M, Wong AC, Herbert AS, Obernosterer G, Mulherkar N, Kuehne AI, Kranzusch PJ, Griffin AM, Ruthel G, et al: Ebola virus entry requires the cholesterol transporter Niemann-Pick C1. Nature 2011, 477:340-343.
3. Davis EM, Kim J, Menasche BL, Sheppard J, Liu X, Tan AC, Shen J: Comparative Haploid Genetic Screens Reveal Divergent Pathways in the Biogenesis and Trafficking of Glycophosphatidylinositol-Anchored Proteins. Cell Rep 2015, 11:1727-1736.
4. Moder M, Velimezi G, Owusu M, Mazouzi A, Wiedner M, Ferreira da Silva J, Robinson-Garcia L, Schischlik F, Slavkovsky R, Kralovics R, et al: Parallel genome-wide screens identify synthetic viable interactions between the BLM helicase complex and Fanconi anemia. Nat Commun 2017, 8:1238.
5. Blomen VA, Majek P, Jae LT, Bigenzahn JW, Nieuwenhuis J, Staring J, Sacco R, van Diemen FR, Olk N, Stukalov A, et al: Gene essentiality and synthetic lethality in haploid human cells. Science 2015, 350:1092-1096.
6. Lau MT, Manion J, Littleboy JB, Oyston L, Khuong TM, Wang QP, Nguyen DT, Hesselson D, Seymour JE, Neely GG: Molecular dissection of box jellyfish venom cytotoxicity highlights an effective venom antidote. Nat Commun 2019, 10:1655.
7. Mair B, Tomic J, Masud SN, Tonge P, Weiss A, Usaj M, Tong AHY, Kwan JJ, Brown KR, Titus E, et al: Essential Gene Profiles for Human Pluripotent Stem Cells Identify Uncharacterized Genes and Substrate Dependencies. Cell Rep 2019, 27:599-615 e512.
8. Chamousset D, Mamane S, Boisvert FM, Trinkle-Mulcahy L: Efficient extraction of nucleolar proteins for interactome analyses. Proteomics 2010, 10:3045-3050.*

**Second round of review**

**Reviewer 1**

One of my main concerns from the first draft of the manuscript was the lack of use of a stringent FDR to detect statistically robust enrichment of sgRNA following drug treatment.

From the replies attached to this draft it is clear that the application of typical statistical cut-offs would render much of the hits described here as non-significant. From the sgRNA summary Table 2 it's clear that the reason for this is that for many genes there is considerable variability in sgRNA representation with often only 2 of 6 sgRNA being defined as enriched. Thus, many of the Tier 2 genes are in this category. This is unusual for CRISPRn screens where typically sgRNA performance is very consistent for each gene (unlike CRISPRa screens). One reason for this is the use of drug concentrations which are very high. Indeed, in the response to previous comments it is made clear that the authors used IC90-99 concentrations for 3 days followed by a lower concentration. In my experience, using too high a concentration still allows the strongest resistance genes to be detected but generally removes the longer tail of weaker hits. In IC60-70 would have been advisable for this type of resistance experiment and would have resulted in more statistically significant hits and a more consistent sgRNA performance for each gene.

In Table 3 what do the columns '2nd best' refer to? Are these LFC of top 2 gRNA? If so, it speaks to my comments above.

**Reviewer 3**

This paper performs drug resistant CRISPR screens on HAP1 cell lines over 27 anti-cancer drugs, and identified the general mechanisms of drug resistance. One gene, C1orf115/RDD1 was found to induce resistance to multiple drugs, and was confirmed by in vitro and in vivo experiments.

One of the major concern is the statistical analysis of the screening results. Using raw, uncorrected p values as cutoff in Tier 2 & 3 is problematic, since these p values without FDR correction will lead to a large number of false positives. Of course it will miss biologically meaningful hits (mentioned in the author’s response), but this is critical for the downstream clustering analysis and the characterization of functional profiles (especially Fig. 1 d-f and Fig. 2a).


Indeed, several evidences support my suspicion that Tier 2-3 may have a large fraction of false positives. For example, in Fig. 1f, only the top three drugs are similar with each other, and the rest of the drugs are largely uncorrelated, even if their MOA is similar. Another example is Fig. 3b, where  many other hits with smaller p value than ELAV1 exist. These evidences indicate a poorly designed statistical analysis theme, even though many of the hits can be validated (Fig. 2-5).


Another concern is about the statistical analysis of MDR genes (page 14)
— why the log2CPM is set to between 2.4 and 3.3? These thresholds appear to be ad hoc.
— What’s the rationale of calculating the third highest ranking, not the second highest ranking?
— The description of "constrained enrichment test” is unclear: what does it mean by "setting the gene ranking for the top number of drugs”?

**Authors Response**

**Point-by-point responses to the reviewers’ comments:**

Reviewer #1: One of my main concerns from the first draft of the manuscript was the lack of use of a stringent FDR to detect statistically robust enrichment of sgRNA following drug treatment.

From the replies attached to this draft it is clear that the application of typical statistical cut-offs would render much of the hits described here as non-significant. From the sgRNA summary Table 2 it's clear that the reason for this is that for many genes there is considerable variability in sgRNA representation with often only 2 of 6 sgRNA being defined as enriched. Thus, many of the Tier 2 genes are in this category. This is unusual for CRISPRn screens where typically sgRNA performance is very consistent for each gene (unlike CRISPRa screens). One reason for this is the use of drug concentrations which are very high. Indeed, in the response to previous comments it is made clear that the authors used IC90-99 concentrations for 3 days followed by a lower concentration. In my experience, using too high a concentration still allows the strongest resistance genes to be detected but generally removes the longer tail of weaker hits. In IC60-70 would have been advisable for this type of resistance experiment and would have resulted in more statistically significant hits and a more consistent sgRNA performance for each gene.

*RESPONSE: To address this issue of stringency we have reanalysed our data using FDR<0.1 cut-off. 72 resistance genes pass the FDR<0.1 cut-off from 20 drug screens with 7 drugs yielding no significant resistance genes at FDR<0.1. This is now noted in the manuscript. For the significant resistance genes, several of these genes are known anti-cancer drug resistance genes (such as TOP1 from topoisomerase I inhibitors; TOP2A from topoisomerase II inhibitor; DCK from nucleoside analogues and SLC19A1 from methotrexate etc.). We also identified and validated some novel resistance genes (PPP6C from docetaxel; NAA60 from methotrexate; YBX1 from cisplatin and SLC43A2 from oxaliplatin) that were found to be significant in our screen. While we have also validated genes that do not meet the FDR<0.1 cut-off confirming these genes as bona fide hits, we appreciate the reviewers concerns and have revised our manuscript to communicate this. Together we are confident that our FDR<0.1 cut-off data set is both stringent and relevant, and we hope that a revised manuscript using only FDR<0.1 cut-off will be acceptable. Most importantly, our screening resulted in the identification of C1orf115/RDD1 as a new factor controlling cell death in response to multiple drugs both in vitro and in vivo. There is significant competition to publish this new drug resistance gene RDD1, which has also been identified as a major resistance gene for an unrelated chemotherapy drug (Jason Moffat, Personal communication) and we ask that the reviewers take into account when considering our revised manuscript.*


In Table 3 what do the columns '2nd best' refer to? Are these LFC of top 2 gRNA? If so, it speaks to my comments above.

*RESPONSE: Yes. Although reporting the 2nd best does not necessarily mean that the remaining sgRNA have extremely low LFC, in the revised manuscript we used LFC from median of all sgRNAs in new Table S3 and S4. However this did not alter the MAGeCK scores (e.g. ranking, FDR etc.) as the MAGeCK uses all sgRNAs for calculation.*

RDD1 characterisation - I can't see any WB showing loss of expression with sgRDD1_A and _B - these should be included - preferably in Figure 6d.

*Response: Western blotting RDD1 has proven to be difficult. We tried to blot RDD1 using a commercially available antibody (from Sigma) and we also generated a new RDD1 Fab phage antibody, however this was not successful using a variety of conditions. We have since produced recombinant RDD1 and are now using this to generate a new polyclonal antibody however this will not be ready in time for this publication. As a working antibody for RDD1 (C1orf115) is unavailable, we performed RT-qPCR and showed that the RDD1 mRNA levels reduced in sgRDD1_A and sgRDD1_B KO cells (new Fig. S7a). Moreover, similar results were obtained using siRNA targeting RDD1 in HEK293T cells (new Fig. S7d and e). To further confirm our results, we performed rescue experiments to show that RDD1 itself is necessary for the effect, and not something about the RDD1 locus (in new Fig. S7f). We hope that the reviewer will accept these data as independent confirmation that we have specifically targeted RDD1 and feel these new data improve the quality of the manuscript and thank the reviewer for this suggestion.*

Reviewer #3: This paper performs drug resistant CRISPR screens on HAP1 cell lines over 27 anti-cancer drugs, and identified the general mechanisms of drug resistance. One gene, C1orf115/RDD1 was found to induce resistance to multiple drugs, and was confirmed by in vitro and in vivo experiments.

One of the major concern is the statistical analysis of the screening results. Using raw, uncorrected p values as cutoff in Tier 2 & 3 is problematic, since these p values without FDR correction will lead to a large number of false positives. Of course it will miss biologically meaningful hits (mentioned in the author’s response), but this is critical for the downstream clustering analysis and the characterization of functional profiles (especially Fig. 1 d-f and Fig. 2a).

*RESPONSE: To resolve this concern we revised our manuscript and no longer include in our analysis any data that does not pass FDR<0.1 (i.e. Fig. 1d-f) and this did not change the overall message of our manuscript.*

Indeed, several evidences support my suspicion that Tier 2-3 may have a large fraction of false positives. For example, in Fig. 1f, only the top three drugs are similar with each other, and the rest of the drugs are largely uncorrelated, even if their MOA is similar. Another example is Fig. 3b, where many other hits with smaller p value than ELAV1 exist. These evidences indicate a poorly designed statistical analysis theme, even though many of the hits can be validated (Fig. 2-5).

*RESPONSE: We reanalysed our data and only report data that passes the FDR<0.1 cut-off. The main message of the study is unaffected by this adjustment and we hope this is acceptable to the reviewers.*

Another concern is about the statistical analysis of MDR genes (page 14)
— why the log2CPM is set to between 2.4 and 3.3? These thresholds appear to be ad hoc.

*RESPONSE: This significance threshold is not ad hoc. We first filtered the MAGeCK screen data to genes that had at least five sgRNAs in the GeCKO library, and at least two supporting sgRNAs (“pos.good.sgrna” in Table S4) with enough read coverage in the Control samples. Enough read coverage in this case corresponded to the number of reads being higher than the peak of the density for each drug, which ranged from 2.4 to 3.3 log2CPM across all drugs (A supplementary figure has been included below for further clarification). However, in a revised version of the manuscript we do not include the MDR analysis we developed for this study, and instead we used FDR<0.1 as a cut off for each hit as suggested by both reveiwers.*


*Figure. The read distribution of control samples from each drug screen. The peak of the density (dotted line) for each drug, which ranged from 2.4 to 3.3 log2CPM across all drugs, were used as threshold to ensure enough read coverage for the statistical analysis of MDR genes.*


— What’s the rationale of calculating the third highest ranking, not the second highest ranking?

*RESPONSE: We had tried to analyse MDR effects independently of individual resistance effects in analysis similar to GSEA. Based on the reveiwers concerns, in the new revised manuscript, we no longer include this analysis based on the entire data set in addition to individual resistance data for resistance genes. We limit our MDR analysis to genes that passed FDR<0.1 for each individual test with significance to two or more drugs used to determine MDR.


To better explain the previous analysis we used so the reviewer understands our old approach, to assess significance of our MDR genes we randomly permuted the gene rankings within each drug, and calculated the test statistic under permutation. We repeated this procedure 30 times across the 20,658 genes, and derived an empirical p-value per gene, which we corrected for multiple testing using the Benjamini-Hochberg method ("MDR FDR" in Table S6). This procedure identified a set of 19 putative MDR genes at a significance level of FDR < 0.05 described in our previous version including four genes that were not identified as significantly enriched in any single drug, but were found to be consistently enriched (GHRHR, MRPL10, UBE2R2, and ZNF597).

The above procedure identified genes that were associated with at least three drugs, but did not estimate how many drugs in total the gene is associated with. To attempt to understand this aspect, we performed further MDR enrichment testing under a constrained setting. This constrained setting was motivated by the observation from our simulation study that genes associated with a fixed number of drugs may also be called as significantly associated with a higher number of drugs, e.g. a gene associated with at least three drugs is likely to be found significantly associated with at least four drugs using the testing procedure above. However, under a constrained setting this is much less likely to occur.*


— The description of "constrained enrichment test” is unclear: what does it mean by "setting the gene ranking for the top number of drugs”?

*RESPONSE: We apologize for the confusion. We removed this analysis and used only FDR<0.1 as a means of identifying MDR genes.


However, to clarify this issue for the reviewers, we provide this text:

Firstly, “setting the gene ranking for top number of drugs” means that the observed rankings for those drugs were left unchanged, i.e. not randomly permuted. This testing procedure is a highly conservative test (see simulation study) for which we believe significantly MDR genes are more likely to contain true MDR genes than the unconstrained test.

Out of the 19 MDR genes we described in our previous version, many of them already were significantly associated with individual drugs at the FDR<0.1 level. In addition to these, we identified the MDR genes with consistently high ranking in three or more drugs.

The description of "constrained enrichment test”: The test statistic for the constrained MDR enrichment test is the same as the unconstrained MDR enrichment test, i.e. the third highest ranking of the gene across all drugs. However, when performing permutation to estimate the null distribution for what constitutes what we would expect to observe by random chance, the gene rankings of the top drugs the gene is associated with are not randomly permuted, i.e. are kept as what was observed in the data. That is, if a gene X has its highest rankings in drugs A, B, and C, then when performing the permutation testing, the rankings associated with drugs A, B and C are left unchanged, while the gene rankings of other drugs D, E, etc. are permuted randomly.

We implemented this testing strategy on the 19 MDR genes, explicitly testing for association in at least four drugs, at least five drugs, and so on. This further testing identified 70 individual gene-drug interactions, in particular highlighted genes that appeared associated with a higher number of drugs, e.g. PMAIP1 associated with 11 drugs, and C1orf115 and DCK associated with 5 drugs, and BAX associated with 3 drugs.

Due to the very high similarity of CRISPR profiles among camptothecin, irinotecan and topotecan, we selected only topotecan to represent these drugs, avoiding selection of MDR genes due solely to consistency within this group of three drugs.*

**Third round of review**

**Reviewer 1**

Overall, the paper has been considerably improved following the reviewers' comments and I commend the authors for their revisions and additional work.

I would be happy for publication pending editorial review if the comments below are addressed.


The section in page 7, lines 31-53 which attempt to address mechanistically why REDD1 loss is a multidrug resistance gene is rather superficial and tells the reader very little about mechanism. To explore this properly would require analyses in the KO vs wild-type cells (RNA-seq, cell cycle, proteomics) and likely more complex immunofluorescence studies. IN the absence of these I would recommend removing this section.


Page 3, line 51 - change to 'discovery'

Page 6, line 40 - Table S8 - none of this gene co-dependencies are further analysed (pathway enrichments etc) or discussed in the manuscript - this table and reference to it should be removed unless this is added as it adds nothing to the overall manuscript as it stands.

Page 7, line 20-21 - I would specifically refer to this as a 'rescue experiment' for clarity and (if this is the case) specifically mention that the OE construct has been engineered (I assume a silent mutation) to be resistant to targeting by the RDD1 dgRNA.

Page 7 - Low RDD1 expression and poor prognosis - is the finding of low RDD1 expression in tumours to be expected if it is a resistance gene, and especially as no evidence from sequencing data that it is a tumour suppressor gene? Is there any evidence that the RDD1 ko cells have faster proliferation or any other fitness advantage compared to the wild-type cells? Otherwise, it's not really clear what this actually means.

**Authors Response**

**Point-by-point responses to the reviewers’ comments:**

Reviewer #1: Overall, the paper has been considerably improved following the reviewers' comments and I commend the authors for their revisions and additional work.

I would be happy for publication pending editorial review if the comments below are addressed.

The section in page 7, lines 31-53 which attempt to address mechanistically why REDD1 loss is a multidrug resistance gene is rather superficial and tells the reader very little about mechanism. To explore this properly would require analyses in the KO vs wild-type cells (RNA-seq, cell cycle, proteomics) and likely more complex immunofluorescence studies. IN the absence of these I would recommend removing this section.

*RESPONSE: We have removed this part as requested by the reviewer.*

Page 3, line 51 - change to 'discovery'

*RESPONSE: Changed to “discovery”.*

Page 6, line 40 - Table S8 - none of this gene co-dependencies are further analysed (pathway enrichments etc) or discussed in the manuscript - this table and reference to it should be removed unless this is added as it adds nothing to the overall manuscript as it stands.

*RESPONSE: Removed*

Page 7, line 20-21 - I would specifically refer to this as a 'rescue experiment' for clarity and (if this is the case) specifically mention that the OE construct has been engineered (I assume a silent mutation) to be resistant to targeting by the RDD1 dgRNA.

*RESPONSE: We clarified the rescue experiment using gRNA-resistant RDD1 construct and mentioned in M&M section.*

Page 7 - Low RDD1 expression and poor prognosis - is the finding of low RDD1 expression in tumours to be expected if it is a resistance gene, and especially as no evidence from sequencing data that it is a tumour suppressor gene? Is there any evidence that the RDD1 ko cells have faster proliferation or any other fitness advantage compared to the wild-type cells? Otherwise, it's not really clear what this actually means.

*RESPONSE: In this section, we used the unbiased data from public dataset to show that low RDD1 expression was associated with poor prognosis in multiple cancers. We compared the proliferation rate of RDD1 KO cells and the wild-type cells, but there is no significant different between two cells. It will be interesting to identify the role of RDD1 in cancer development and progression in future studies.*
